# Refining Our Understanding of Howiesons Poort Lithic Technology: The Evidence from Grey Rocky Layer in Sibudu Cave (KwaZulu-Natal, South Africa)

**DOI:** 10.1371/journal.pone.0143451

**Published:** 2015-12-03

**Authors:** Paloma de la Peña

**Affiliations:** Evolutionary Studies Institute, University of the Witwatersrand, Johannesburg, South Africa; Universidade do Algarve, PORTUGAL

## Abstract

The detailed technological analysis of the youngest Howiesons Poort occupation in Sibudu Cave, layer Grey Rocky, has shown the importance of blade production (with different knapping methods involved), but also of flaking methods in coarse grained rock types. Moreover, new strategies of bifacial production and microlithism were important. Grey Rocky lithic technology shows a really versatile example of reduction strategies that were highly influenced by the characteristics of the rock types. This lithic assemblage is another example of the technological variability linked to the Howiesons Poort technocomplex. The reasons for this variability are still difficult to elucidate. Discrepancies between sites might be for different reasons: diachronic variations, functional variations, organizational variations or maybe different regional variations within what has been recognized traditionally and typologically as Howiesons Poort. The technological comparison of the Grey Rocky assemblage with assemblages from other Howiesons Poort sites demonstrates that there are common technological trends during the late Pleistocene, but they still need to be properly circumscribed chronologically. On the one hand, Howiesons Poort characteristics such as the bifacial production in quartz are reminiscent of production in some Still Bay or pre-Still Bay industries and the flake production or the prismatic blade production described here could be a point in common with pre-Still Bay and post-Howiesons Poort industries. On the other hand, the detailed analysis of the Grey Rocky lithics reinforces the particular character of this Howiesons Poort technocomplex, yet it also shows clear technological links with other Middle Stone Age assemblages.

## Introduction

### Howiesons Poort in Retrospective

Howiesons Poort (HP) is one of the better known industries of the southern African Middle Stone Age sequence. However, although it is well known, it is not well-understood. Depending on the researcher concerned, it has been named an industry [1–3], a horizon marker [4], a Middle Stone Age (MSA) entity [5] and recently a technocomplex [6] or a techno-tradition [7], following the mainstream technological approach. The two last terms are presently the most common ones in the MSA literature. The term ‘technocomplex’ was formulated by Clarke [8] referring to a set of cultures having a number of common features. In Western European studies, this term is also used by the practitioners of the G. Laplace typology. However, nowadays ‘technocomplex’ is used in Palaeolithic studies in another sense, as a result of the relatively recent trend in technological analysis. Thus a technocomplex is seen as synonymous with longstanding technical traditions. It is striking that while the methodology for analysing the industries has radically changed -from a typological approach to a technological approach- the names used to identify the assemblage remain the same and typology still seems the main way to guide the recognition of assemblages. This epistemological trend has also been recognised in Upper Paleolithic technocomplexes in Europe, which were formerly identified as cultures, then as typological traditions, and more recently as technocomplexes. The best example is maybe the Gravettian and its multiple *facies* [9]. HP has followed a similar historiographical evolution, from a cultural typological conception to a technocomplex.

This paper follows a technological approach and explores specifically the lithic-technological definition of HP, putting the stress on the knapping methods and the management of different rock types as a *proxy* for understanding the organizational and economical behaviour for the assemblage analysed. In this paper I will relate the knapping reduction strategies found at the most recent HP’s layer at Sibudu with other contexts also identified as HP (either in a typological or in a technological manner) in order to test whether the term ‘technocomplex’ (understood as a longstanding technical tradition) can be applied for different assemblages identified as HP.

Since its first inception, HP has been considered as an unusual industry because of its Later Stone Age/Upper Palaeolithic-like implements and, later on, the focus on the study of HP industries has highlighted a great variety of material culture that is now known to accompany the HP lithic repertoire, such as engraved ochre, bone technology and ostrich eggshell engravings [10–13].

In the first half of the XXth Century researchers such as Goodwin, using an evolutionary perspective, proposed that the advanced material culture demonstrates that HP corresponds to the final MSA or transition between the MSA and LSA (a similar hypothesis has lasted until recently, see [14]).

In the Third PanAfrican Congress on Prehistory (1955) the HP (together with the Magosian) was included in the so called ‘Second Intermediate period’ for the MSA, as a transitional industry between the MSA and the LSA. The basis of this was mainly typological, on the presence of backed and/or truncated pieces manufacturated on fine-grained materials [15]. Afterwards, the Magosian turned out to be a fallacious lithic tradition (because the site where it was defined was a mixed assemblage) whereas the name and concept HP remained intact.

Later on Singer and Wymer [16] used population replacement as an explanation, that is, the makers of ‘traditional’ MSA tools were replaced by a new population creating HP tools, and that the original southern African inhabitants returned to their homeland after the demise of the HP. Besides this interpretation, it is important to highlight that the Singer and Wymer’s excavations at Klasies River Mouth confirmed that HP was an industry in the middle of the MSA sequence and not a late MSA industry, as typological proposals suggested. This was later confirmed in sequences such as Rose Cottage [17], Umhlatuzana [18], Border Cave [19] and Apollo 11 [20].

Deacon [4, 21] proposed that lifeways implied by material culture associated with the HP could be compared to the San ethnographic record. The environmental stress generated by climatic deterioration at the beginning of the HP stimulated the creation of new social adaptations; and backed pieces would have been a way to mark social identity between groups, with social interchange system such as the *hxaro* gift-giving partnership system of the Kalahari San described by Wiessner [22]. For Deacon this social trend would also have been a sign of ‘modern behaviour’.

Wadley [23]:90–91 compared the HP with the Wilton, suggesting that both could be result of similar technological responses to similar demographic, social and economic conditions. She also pointed out how the Stone Age had fluctuating forms of social relations and the Wilton and HP may represent related, yet different forms.

Indeed, HP has been one of the main archaeological entities enabling a claim that ‘modern human’ behaviour was present in the MSA, and that behaviour in the MSA was not that different from behaviour at the beginning of the LSA or the so-called ‘Upper Palaeolithic revolution’ [24].

Nonetheless, the archaeological characteristics associated with HP and conceived as ‘advanced’, such as the blade technology [25], symbolic behaviour [26], etc. have been sometimes presented as ephemeral e.g. [27]. In other words, behaviour in the HP was viewed as an anomaly within the general trend of development within the MSA.

Finally, among the different propositions, it must be highlighted that harsh environmental conditions were also used in order to explain HP development [21, 26, 28–30].

### Technology and Howiesons Poort

The reality is that, regardless of efforts to define the HP technologically, it is still usually recognized typologically. This is a problem which it shares with the SB, which is also mainly identified by typology. This seems paradoxical as HP and SB are by far the most studied and cited archaeological entities in the South African MSA.

From a typological point of view Thackeray [31] gave an accurate definition of southern African HP assemblages: ‘*southern African MSA stone artifact assemblages characterized by various backed and/ or truncated pieces such as trapezoids and segments (crescents)*, *often considered larger than those found in Later Stone Age assemblages (…)*, *which are often made on fine-grained raw materials such as silcrete and chert*. *These artifacts are found in addition to typical Middle Stone Age flake-blades (elongated flakes) and flake-blade sections*, *a negligible proportion of which is retouched to form unifacial and bifacial points*, *denticulates*, *or scrapers*’ [31]: 390.

In the last fifteen years, typological criteria have been supplemented by HP technological definitions in different regions of South Africa. Furthermore, there has lately been a notable effort to define the entire technology of the HP. Nevertheless, the technological definition of the HP requires a lot more research, notwithstanding that the work of Wurz [25] on the Klasies River assemblage began to change the definition of this industry from a typological towards a technological one. Wurz [25] proposed that HP blades originated from a recurrent blade production system using a soft hammer. She also proposed that this blade production was the basis for creating the blanks for the backed tools in Klasies River HP layers (in other HP assemblages, however, flakes are the most used blanks in order to produce backed implements, e.g. for Diepkloof, see Mackay [32]).Indeed, variants of HP have been identified at sites such as Umhlatuzana, Rose Cottage, Sibudu, Diepkloof and Klipdrift [6, 18, 25, 33–39].

A relevant example for the technology of HP is Rose Cottage. In this site the blade production and the formal tools have been profusely described [17, 33, 38]. In the HP layers of Rose Cottage knapping strategies seem really constrained by the rock type chosen: almost exclusively opaline cryptocrystalline nodules. Soriano et al. [33] compare the knapping strategies with opaline nodules to produce bladelets from the HP of Rose Cottage with the blade production in the Chatelperronian in France, which represents a break with Middle Paleolithic blade production. They also emphasize the importance of ‘marginal percussion’ among the techniques of knapping associated with the HP. Moreover, in their study they distinguish between a ‘‘classic” HP, which corresponds to the base and the middle of the HP sequence. In these layers (layers EMD and MAS), blade production is carefully carried out with very marginal percussion. The second phase, or final HP (layers ETH and SUZ), is marked by a degradation in the quality of blade production, observable in the lower degree of regularity in products and a less elaborate platform preparation. Furthermore, in this second phase, marginal percussion disappears.

One of most recent studies of HP technology is the one of Diepkloof. In this site the HP industry was defined as a regionally specific technocomplex that, unlike other HP industries, could be subdivided into phases (Early, Intermediate and Late) [6]. Moreover, in their study they propose that there is interstratification of a non-HP lithic technology between the Early and the Intermediate HP, the so called MSA type ‘Jack’, even though this layer contains large backed pieces which, traditionally have been the hallmark for typological recognition of HP assemblages. Porraz et al. [6] see similarities, in terms of technological strategies, among the three phases of HP (such as the ‘HP core reduction’). Nevertheless in the Late Phase they point out an increase of irregular blades coupled with an increase in flake production. In other words, between the Early, Intermediate and Late HP that they define they observe the same *chaîne opératoires*. Therefore, the variations that they point out between these three phases are mostly related to the formal tool repertoire, with mainly *pièces esquillées*, truncated pieces and bifacial pieces in the Early HP; strangulated pieces and notched pieces in the Intermediate HP and, finally, mainly backed pieces in the Late HP. They state that the significance of these three distinct HP phases cannot be functional and that the comparisons with other HP sites suggest that these three phases represent a technological trend within HP. Furthermore, after their analysis they propose that in most of southern African sites the industries identified as HP probably correspond to the Intermediate and Late phase from Diepkloof that they name ‘classic’ HP (a terminology previously used by Soriano et al. [33], but with a slightly different definition, *vid*. *supra*).

At Klipdrift the conclusion of the technological analysis was that three phases within the ‘HP complex’ could be distinguished. The lowermost phase (layers PCA, PBE) is characterized by silcrete as the main rock type and, typologically, by the presence of notched pieces, strangulated pieces and highly standardized truncated blades. The intermediate phase (PBC, PBA/PBB) is characterized by an increase in the quartz exploitation and by the increase of backed pieces among the retouch tool kit. The final most recent phase (PAY layer) is characterized by the use of quartzite and by an increase of the size of the blade production, together with an increase of flake knapping methods (such as *Levallois*). Henshilwood and colleagues propose that this phase could be interpreted as a ‘transitional layer’ towards post-HP. The common trend in these three phases is that the knapping reduction sequence is almost entirely devoted to the production of blades [39].

In two recent studies by de la Peña and Wadley [36, 37], one of the HP layers of Sibudu was studied in depth from a technological point of view: Grey Sand (which has an age estimate of 63.8 ± 2.5 ka, [40]). In these two studies, new knapping methods were recognised in the HP, such as a well-developed prismatic technology for big blade production (for dolerite and hornfels), varieties of core on flakes for bladelet production (for dolerite and hornfels), and strategies of microlithism for quartz reduction, including an extensive use of prismatic cores and, subsequently, bipolar knapping in order to produce microliths (bladelets and small flakes). Moreover, recent technological studies at Diepkloof and Sibudu have highlighted bifacial reduction sequences within HP assemblages [6, 41].

The recent technological analysis of a sample of Sibudu, Rose Cottage and Klasies River assemblage have highlighted several technological trends for the HP [35]. They assign all these HP assemblages to the so-called ‘classic HP’ defined at Diepkloof (this means the Intermediate and late HP phases of Diepkloof site). For Sibudu different technological trends are pointed out, such as soft stone hammer percussion for the flaking techniques and an axial hafting for backed implements. Furthermore, this study highlights the similarities in blade knapping methods between Klasies and Sibudu, this is proposed from the morphology of the blades and the same frequencies in these two assemblages. This recent study considers HP as an ‘industry’, identified with a ‘cultural entity’, and its coherence within South Africa, in terms of technological and typological trends. This is stressed in contraposition to SB assemblages.

As a result of these new technological analyses, variation in HP industries is beginning to be recognised. In other words, it no longer seems to be the homogeneous lithic phenomenon promoted by the former typological approach.

It seems that one of the main challenges for constructing a better definition of HP is first to establish the meaning of variability in the context of this industry, thereby enabling the distinction between diachronic/chronological, functional, organizational and regional technological variation.

Together with lithic technology studies, ochre and residue analyses, macrotrace use wear analyses, and experimental approaches have pointed to evidence for varied hafting strategies for composite tools, using backed HP implements, first in Sibudu Cave [42–45], and later on in Diepkloof [46]. Such technological strategies (which are related to lithic technology and which complement them) might, in the future, give us different perspectives on technological variation within HP assemblages.

### The Chronostratigraphical Position of Howiesons Poort

The HP chronology is currently the subject of intense scientific discussion. Jacobs et al. [3] proposed a short time frame for HP and SB industries based on the evidence from nine southern African sites. In that study Jacobs et al. [3], using single-grain optical luminescence dating (OSL), proposed that HP and SB were short-lived (5000 years or less) industries separated, at the sites that were dated, by about 7000 years. They proposed that HP started around 64.8 ka and ended 59.5 ka, with duration in the timeframe of about 5.3 ka [3]. Within this short-lived model, HP belongs to Marine Isotope Stage (MIS) 4. However, Tribolo et al. [47] have challenged the model in recent work at Diepkloof, based on TL and OSL dating of 39 burnt lithics and 5 sediment samples. The results are significantly older than those reported by Jacobs et al. [3]. They obtained a chronology of 105 ± 10 ka and 109 ± 10 ka for the Early HP; 77 ± 8 ka, 85 ± 9 ka, 83 ± 8 ka 65 ± 8ka for the Intermediate HP and 52 ± 5 ka for the Late HP. Therefore, the time span proposed for the three technological phases related to HP in Diepkloof is between 109 and 52 ka ago [47]. Thus, in this new model, HP first appears in MIS 5 and disappeared in MIS 3. Feathers [48] reanalysed samples from HP layers of Diepkloof and Kathu Pan 6 and has produced slightly older or equivalent dates to those provided by Tribolo et al. [47]. Moreover, Jacobs and Roberts [49] have reanalysed and reassessed their sediment samples from Diepkloof to address some of the criticisms of their Diepkloof chronology [3]. In this new research they obtained ages that are robust and consistent with their original chronology, but they cannot satisfactorily explain why the TL and OSL ages provided by Tribolo et al. [47] are different. They do point out that their sediment samples were not collected from the same part of the Diepkloof excavations as samples collected by Tribolo and Feathers. In conclusion, regarding the HP chronology, the debate remains open.

The controversy around HP chronology presents a major problem for two reasons: on the one hand, for discussions of technological variability through time and, on the other hand, for the correlation of HP technology with climatic models. Moreover, the uncertain chronology makes it difficult to understand what HP represents. There is a big difference between a techno-tradition that is a ‘short lived’ cultural phenomenon (5ka) as opposed to a lithic tradition lasting 45-50ka.

## Objective

In this paper I present an in depth technological study of the youngest HP layer in Sibudu Cave: Grey Rocky (GR). This is a rich layer with optimal organic preservation [50]. Previous lithic analyses of part of Wadley’s collection and GR have been also published [14, 35, 51].

In a preliminary study it was noticed that flake reduction strategies might be present, together with other types of blade knapping methods. Moreover, bifacial reduction on quartz has been documented and explained in a previous work [41]. In other words, this layer is particularly interesting because, together with the typical techno-typological elements associated with HP (backed tools, profuse blade production), it was already known that other specific technological traits were present. Therefore, the main aim of describing GR technologically is to explore HP lithic variation as it occurs in this eastern part of Southern Africa. Sibudu remains the main sequence for this part of the continent, not least because of its chronostratigraphy.

The main objectives of this paper are three: first, to give a detailed technological description of the youngest HP occupation in Sibudu Cave, making use of all the lithics excavated from six square metres during Wadley’s campaigns (note that the recent paper of Soriano et al. [35] only took into account a sample of this layer). Secondly, I shall establish a strong methodological protocol (qualitative attribute analysis and basic statistical tests) that in the future will be used to tackle technologically other MSA lithic assemblages in Sibudu Cave and other HP sequences.Thirdly, to place the results of this technological study in a broader discussion around HP and the MSA variability.

### Sibudu Rock Shelter and the Layer Studied

**Fig 1:**
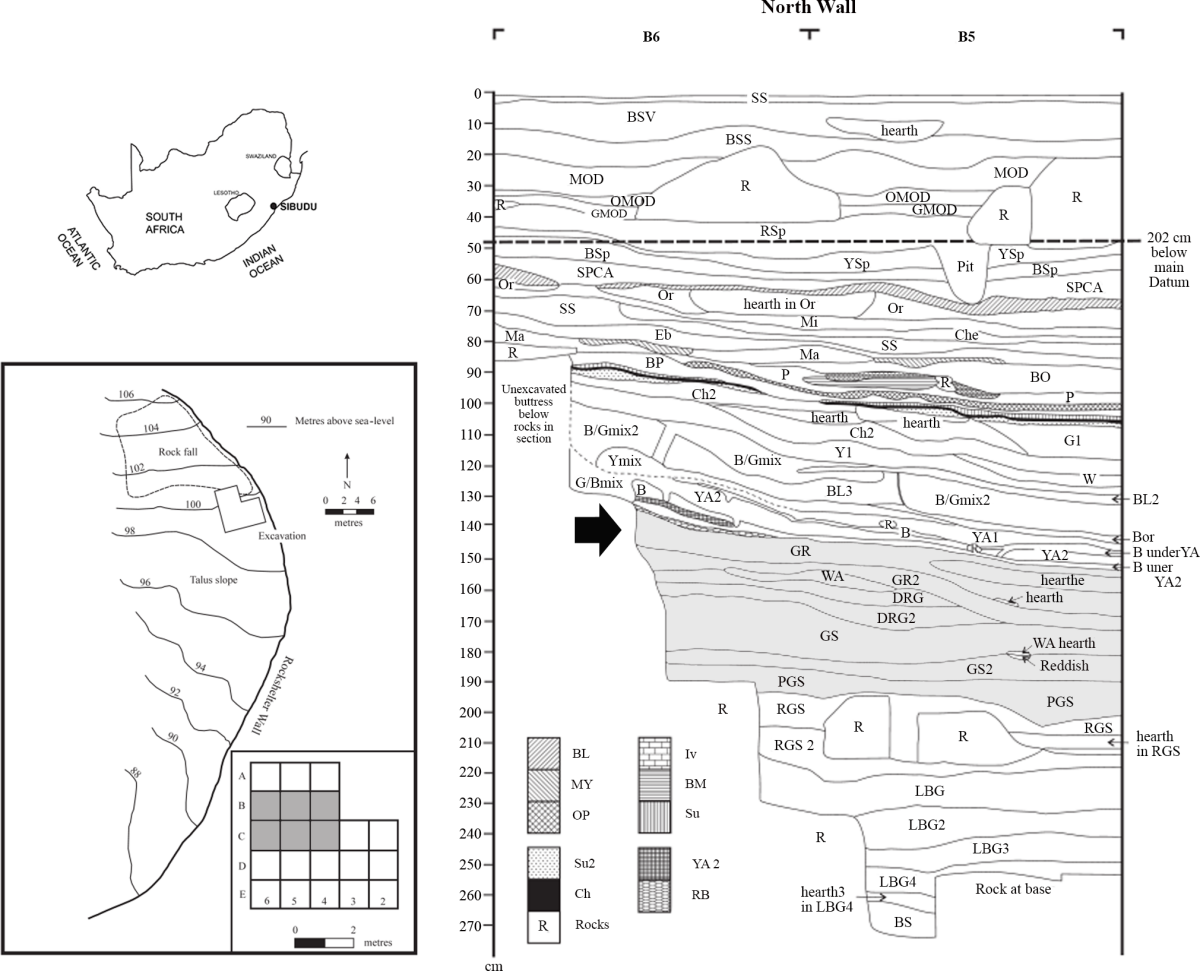
Location of Sibudu Cave. On top left, location of Sibudu Cave (29.522627S, 31.085895E). On the bottom left, Plan of Sibudu Cave. This schematic map was made on the basis of a topographic map of Southern Africa, source: Maps at the CIA (public domain): https://www.cia.gov/library/publications/the-world-factbook/index.html. On the bottom left the excavation grid is represented with the square meters for this analysis highlighted in grey. On the bottom right, stratigraphy of the North wall of Sibudu Cave (Stratigraphy courtesy of Lyn Wadley). The HP layers are highlighted in grey.

Sibudu is located approximately 40 km north of Durban, and about 15 km inland of the Indian Ocean, on a steep cliff overlooking the uThongathi River (29.522627°S, 31.085895°E) (Fig 1). The shelter is 55 m long and 18 m in breadth and has a long occupation sequence with several layers and features corresponding to the pre-SB, SB, HP, post-HP, late MSA, final MSA and Iron Age [52].

HP occupations reported here come from six square metres (squares B4, B5, B6, C4, C5 and C6) of Wadley’s excavations in the deep sounding.

The layers associated with the HP are (from the base to the top): Pinkish Grey Sand (PGS), Grey Sand (GS, GS2 and GS3), Dark Reddish Grey (DRG) and Grey Rocky (GR and GR2) (Fig 1) [52].

**Fig 2:**
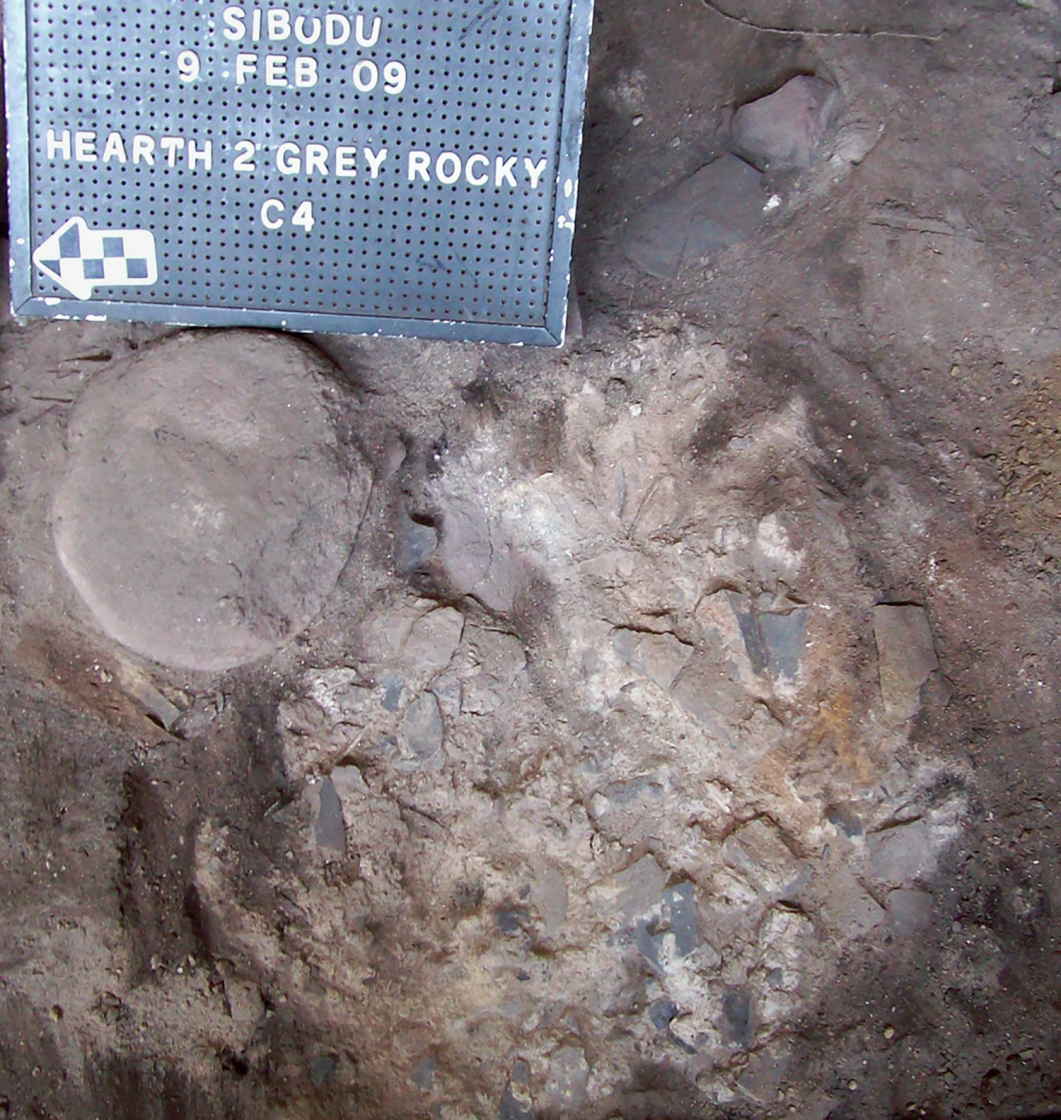
Hearth 2 in GR layer, square C4. Picture from L. Wadley’s excavation. Photograph courtesy of Lyn Wadley.

The stratigraphy is clear, combustion features are discernible [50, 53](Fig 2), and micromorphology implies that most Sibudu layers have stratigraphic integrity [54], although rock fall between the oldest HP layer, PGS, and the underlying SB layer, Reddish Grey Sand (RGS), has caused some disturbance.

The layer that I discuss here is GR which is light, brownish-grey silt. In B6 the layer GR is in between a rock fall. GR2 is an artificial spit to divide the layer. During the excavation several features within GR were distinguished (Table 1) [50, 53]; as can be seen there are abundant hearths (Fig 2). The chronology of GR layer is 61.7±2 Ka obtained from single grain optically stimulated luminescence on sediment from GR2 [52].

**Table 1:** List with the different stratigraphic features recognised as part of GR and GR2 in Sibudu, from Wadley’s excavation.

Grey Rocky under Hearth 1
Grey Rocky under Hearth 2
Grey Rocky under rock
Hearth 1 in Grey Rocky
Hearth 2 in Grey Rocky
Hearth 3 in Grey Rocky
Hearth 4 in Grey Rocky
Hearth 5 in Grey Rocky
Hearth A in Grey Rocky
Hearth B in Grey Rocky
Hearth C in Grey Rocky
Hearth D in Grey Rocky
Hearth E in Grey Rocky
Hearth 1 in Grey Rocky 2
Hearth 2 in Grey Rocky 2 (black base)
Hearth 3 in Grey Rocky 2
Hearth A in Grey Rocky 2
Hearth B in Grey Rocky 2
Hearth C in Grey Rocky 2
Hearth D in Grey Rocky 2
White ash below Grey Rocky 2
White ash under Grey Rocky 2
Yellow Ash in Heart E in Grey Rocky 2

## The Rock Types Knapped at the Site

**Fig 3:**
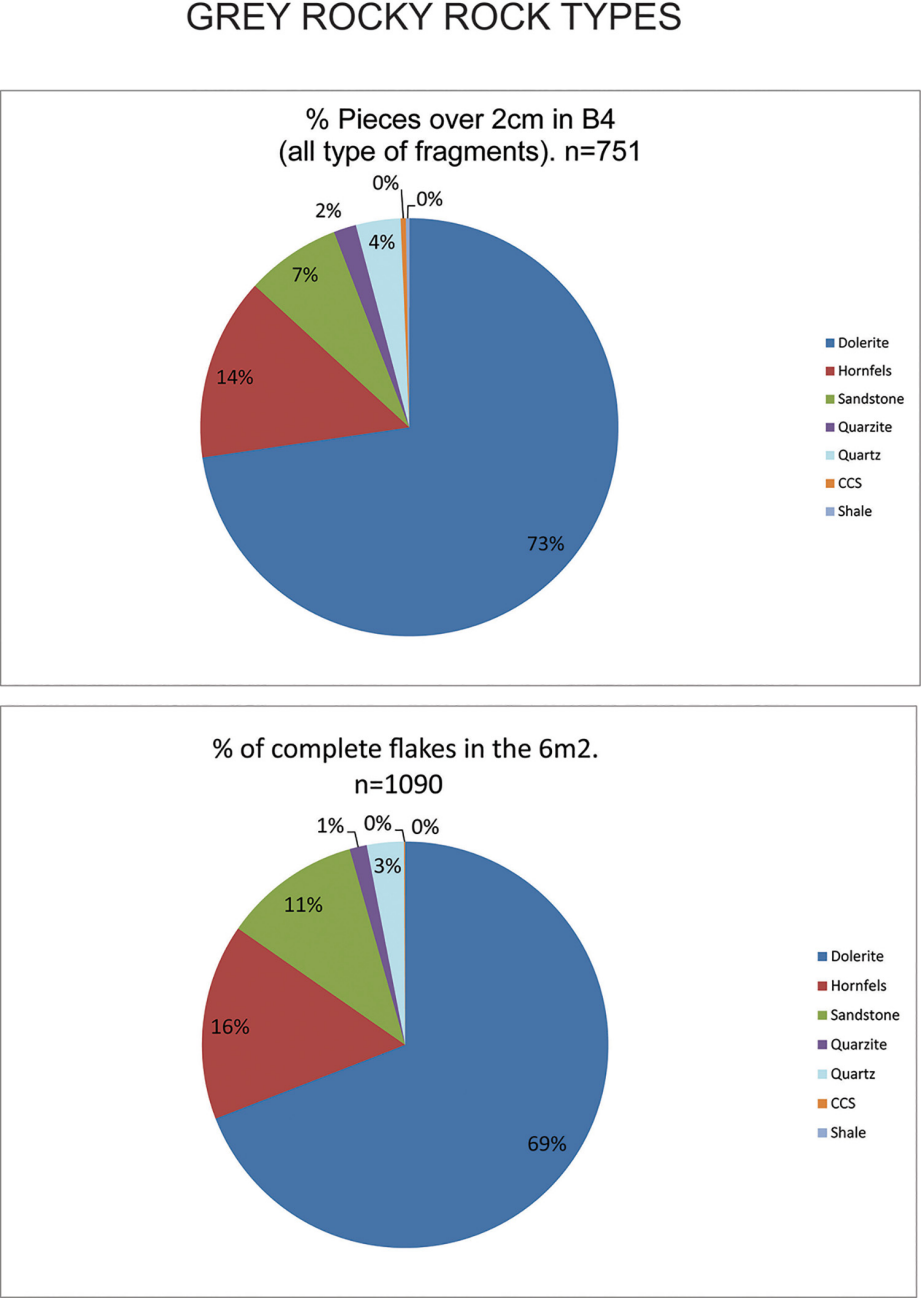
Percentages of rock types. Top: percentage of rock types in Sibudu’s layer GR amongst pieces over 2cm in square B4. Bottom: Percentage of rock types for complete flakes in the 6m^2^ analysed (see S1 File). As can be seen these two samples are very similar and the main rock types knapped in GR layer at Sibudu are: dolerite, hornfels, sandstone, quartz, quartzite and cryptocrystalline material (in order of percentage representation in the layer).

As shown in Fig 3 the main rock types and minerals knapped in the GR layer at Sibudu are: dolerite, hornfels, sandstone, quartz, quartzite and cryptocrystalline material (in order of percentage representation in the layer).

The uThongathi River below Sibudu is a source of weathered and river-rolled dolerite and quartzite as well as of small quartz pebbles. The rounded cortex on a number of Sibudu cores and flakes implies a waterborne origin for some dolerite brought to the site, but cores and flakes made from tabular dolerite pieces are also present. Abundant igneous dolerite near Sibudu derives from intrusive Jurassic volcanism, mostly as sills, although a true dolerite dyke lies close to the rock shelter and this seems likely to have been the source of much of the dolerite at the site. Dolerite sills in the area include fine-grained ones like the Mhlasini sill and coarse-grained ones like the Verulam sill [55]. Petrographic analysis of a thin section of fine-grained dolerite showed that the minerals include 45% clinopyroxene, 44.5% plagioclase and small percentages of quartz, limonite and goethite [56]. Unfortunately, dolerite is chemically similar across large regions of South Africa and cannot be distinguished, although the Effingham sills in KwaZulu-Natal have higher silica contents (in excess of 63 wt%) than most of the others.

Where dolerite intrudes into shale, there are bands of metamorphic hornfels. Differing temperatures occur in the zone of thermal metamorphism where a dolerite intrusion occurs. Consequently, there are different grades of both hornfels and dolerite. Hornfels is difficult to find in the Sibudu area, but one source is near Verulam, about 15 km from Sibudu. This hornfels is poorly metamorphosed, and occurs as thin slabs, only a few centimetres thick. XRF elemental analysis of a piece of hornfels from Sibudu demonstrated its high silica (63.8%), low magnesium (1.4%) and calcium (0.7%) content (relative to that of dolerite) which confirms that it is metamorphosed shale from a contact zone with a dolerite intrusion [55]. A survey along the uThongathi River by Helen Kempson in 2011 showed that hornfels nodules are not present there [56]. Moreover, it must be taken into account that the landscape might have changed a lot since the HP, and available outcrops in the past might not be visible now.

Sandstone is the ‘local’ rock type at the site, as the Sibudu rock shelter is situated in a sandstone cliff within the Mariannhill Formation of the Natal Group [55]. The sandstone knapped at the site looks identical to the one that is forming the rockshelter. It is quite a coarse grained subtype. The granular structure of this rock makes it a suitable material to knap.

Crystalline quartz appears as river pebbles and as pebbles inside conglomerates in the Verulam area [55]. Some big quartz pieces could come from river pebbles but this does not seem the case for some other big pieces. Probably quartz dykes were used as a source for this raw material in the past, judging by the macroscopic characteristics of some cortex areas of these pieces. Within the crystalline quartz assemblage two main categories can be distinguished and both were exploited in the HP layers of Sibudu [37]: vein quartz (milky or xenomorph) and crystal quartz (also called hyaline or automorph quartz) [57, 58]. As pointed out by de Lombera-Hermida [58]:102 crystalline ‘quartz formation processes must be taken into account in order to establish a good petrological classification and characterization’, and these characteristics have important implications for the mechanical properties of different quartz varieties. Xenomorph quartz displays greater variability because of different chemical and physical causes of its formation process [58]:102. All these conditions generate different types of crystalline quartz and, therefore, different types of mechanical properties. Martínez and Llana [59] made a morphostructural classification taking into account grains and planes (flaws or crystalline surfaces) of crystalline quartz. Four morphostructural groups were distinguished: NN: no grain, no plane; NS: No grain, plane; SN; grainy, no plane; SS: grainy, plane. Following this classification Sibudu’s vein quartz is NS in layer GR. In addition, as pointed out in previous publications, hyaline or crystal quartz was also knapped [37, 60].

Cryptocrystalline or microcrystalline quartz also appears within conglomerates in this area [55] and it is one of the minority minerals knapped in this layer. Cryptocrystalline minerals in GR are really fine grained and they have very well developed conchoidal fracture patterns.

Quartzite appears as river-rolled small pebbles and it is usually medium/ fine grained.

## Methodology

The excavation of Sibudu was conducted with a permit obtained from Amafa i KwaZulu-Natali, the Heritage Agency based in Pietermaritzburg, South Africa. No ethics clearance or permit is required to study the lithic artefacts from Sibudu.

This study employed the *chaîne opératoire* approach, with which an assemblage is viewed as the outcome of cultural choices by a human group or several human groups that occupied this rock shelter more than 60 000 years ago.

The *chaîne opératoire* approach assumes that technological choices are ordered and can be classified into different stages [60–63], from the selection of the rock types (acquisition) to the discard or recycling after use [62]. Having said this, I acknowledge that this methodology is a systemic approach. In other words, one is forced to consider the assemblage of lithic blanks as a whole, despite the fact that they were likely produced by different human groups at different times. Therefore, the systemic nature of the *chaîne opératoire* can sometimes provide a skewed perspective. In other words, analysing all the lithics from GR through the *chaîne opératoire* approach means that I must assume that they are technologically related and can be connected; and that they always share some sort of technological affiliation. There are limitations to this approach, because to demonstrate the assumption that all the lithics are related and part of the same system, refitting analyses should be undertaken. This, however, would be extremely difficult for the Sibudu materials because of the similar colour and other macroscopic characteristics of the lithic assemblage’s raw material types, and because the size of Wadley’s excavation (6m^2^) is only about 2% of the roughly 300 m^2^ of the Sibudu deposit. Furthermore, as different scholars have pointed out [64–66], the *chaîne opératoire* approach can be highly subjective. In order to make this study as objective as possible, and in order to allow the GR assemblage to be compared with other Sibudu and MSA assemblages, I incorporated various quantitative parameters in an attempt to limit subjectivity that may arise from a purely qualitative and systemic approach. Furthermore, after the attribute analysis, I applied different basic statistics to support the qualitative arguments.

The sample studied from GR was the following: all cores (n = 120), core related by-products (n = 63) and retouched pieces (n = 244) from the Wadley’s excavations. In order to understand lithic production better I also analysed all complete flakes (n = 1091) (see S1 File). Finally, all lithic artefacts (including chips) were examined in order to identify and analyse any items of potential technological importance. The lithic assemblage was divided into four broad analytical categories: (1) cores, (2) blanks without retouch, (3) retouched blanks and (4) chips. The chips include pieces with a wide range of morphologies that are smaller than 10mm for quartz, since previous studies of the GS layer showed that this cut-off was the most appropriate for this material [36, 37], and smaller than 20mm for the other raw material types. Moreover, I examined all the lithic remains, including chips in a preliminary analysis of the study of GR. In that analysis I separated all the retouched pieces even when they were under 10mm and was able to identify morphotypes such as bifacial pieces, small notches and backed pieces, especially with respect to quartz pieces.

Cores and core-related by-products can provide important qualitative information about knapping methods, although, as previous researchers have also pointed out [64] they can provide a limited view of the reduction sequence. Indeed, cores usually reflect only the latter stages of reduction and discard. An understanding of the knapping process results from a quantitative and qualitative study of the majority of the blanks, especially those from flake production [67].

**Fig 4:**
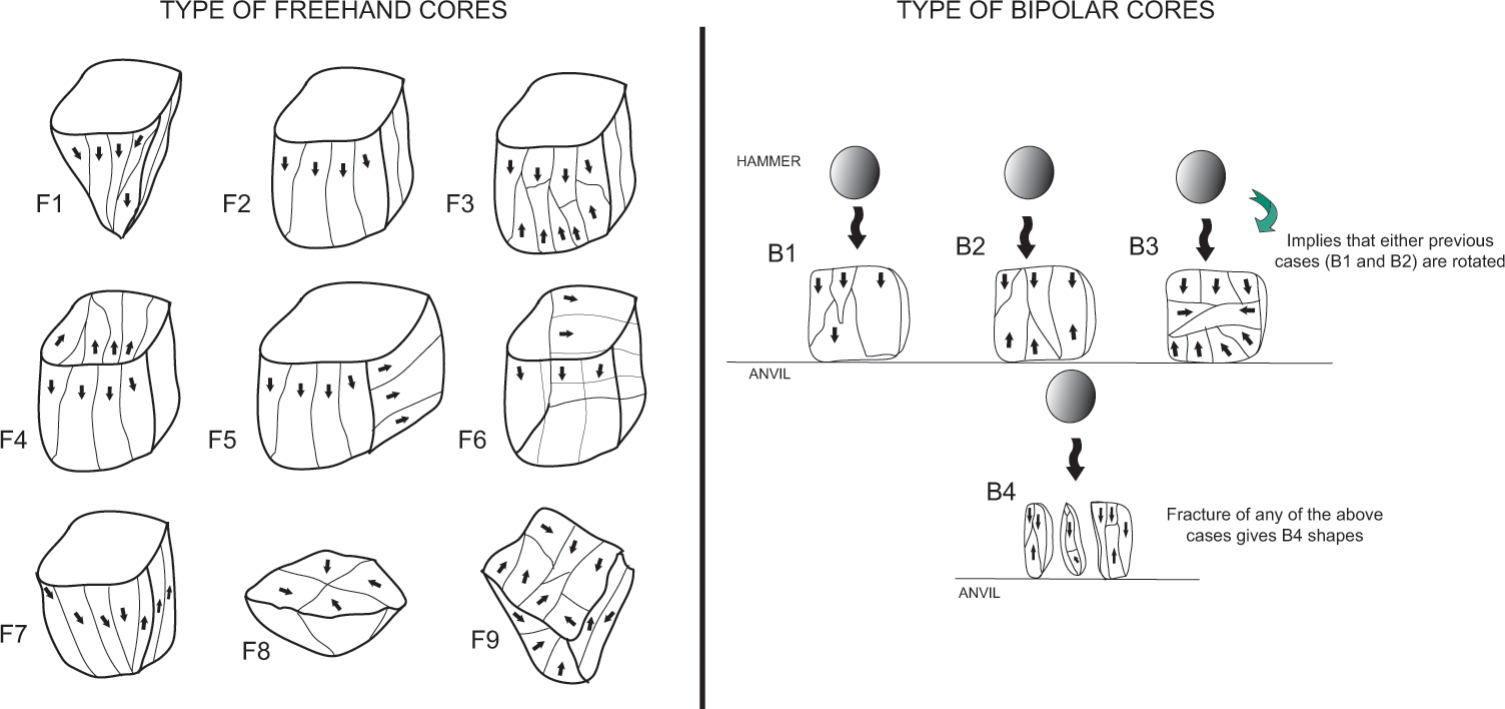
Freehand and bipolar quartz cores classified in this analysis. Left: types of freehand cores. F1 conical core; F2, F3, F4, F5, F6 and F7 various types of prismatic cores (with different directions of removals); F8 centripetal core; F9 multifacial core. Right: different types of bipolar cores recognised in this study, with different directions of removals. B1. Unidirectional. B2. Bidirectional. B3 is the result of rotation. B4 is typical of fracture accidents during bipolar knapping.

**Fig 5:**
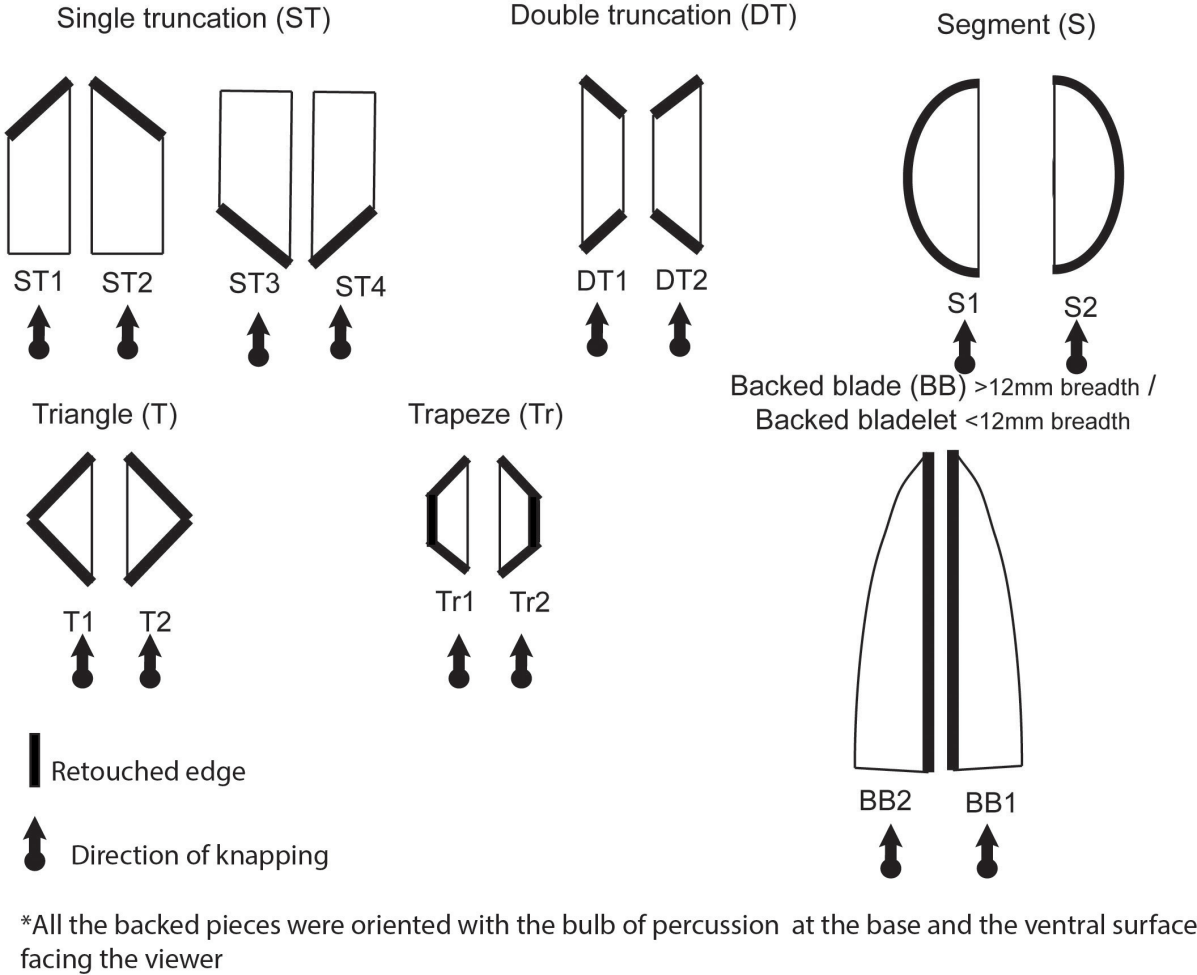
Different types of backed pieces considered in this study. This typological classification takes into account the lateralization of the retouch and its curvature or lack thereof. The single trunctation type is what, in the southern African literature, is referred to as an ‘oblique backed point’

The specific lithic attributes recorded are described in Tables 2–5 and in complementary Figs 4 and 5. Most of the qualitative variables for the hornfels and dolerite core study come from the Pelegrin method for Chatelperronian cores [63]. The study of cores on flakes follows previous technological and experimental work, such as [68, 69]. The study of Sibudu’s quartz cores uses the attributes presented in previous quartz studies, such as [70–71] and my own experimentation criteria [72], in order to distinguish between freehand and bipolar knapping. Furthermore, in this study I used the categories proposed for HP cores in two previous technological papers on Sibudu’s HP layer GS: one focused on quartz [37] and another examined knapping methods from core morphology [36]. Such consistency should facilitate future comparisons. It must be clarified that the term ‘core on flake’ refers here mainly to *Kostienki* cores, burin-like cores and end-scraper cores. Within category 2 (blanks without retouch) I distinguish:

(2.1.) Flakes. In the study database, it was recorded if a flake was in fact what may be technologically identified as a blade ─items whose length is greater than twice its width and with parallel dorsal ridges and trapezoidal or triangular in cross-section;
(2.2.) Core-related by-products. This category includes all blanks that show any characteristic of core morphology (and therefore knapping methods). They could be produced purposefully or accidentally. In other lithic studies this category is referred to as ‘core trimming elements’ [73] and in previous publications they are referred to as ‘trimming or maintenance by-products’ [36]. Within this category, other specific pieces are identified (2.2.1.) as crests or semicrests for the initiation of a core or for core maintenance and (2.2.2.) false crests or semicrests. With respect to the latter, they are not maintenance by-products, but rather result from a change in the direction of blank removal with the crest representing a previous overhang, see [36]:31, and Fig 4 within it, and GR examples below;
(2.2.3.) Cleaning flakes. These are pieces that may result from correcting errors or repairing accidents that affected the striking platform;
(2.2.4.) Flakes exhibiting knapping accidents;
(2.2.5.) Flakes exhibiting core morphology.

**Table 2:** Variables recorded for quartz cores.

Type of blank
Presence of cortex (0, 1–10, 10–40, 40–60,60–90, 90–99, 100%).
Length, breadth, thickness (maximum measurements for all three) and weight
Number of striking platforms
Orientation of striking platforms
Length and breadth of striking platforms (in case of several, the larger)
Type of preparation of the striking platform (simple flake, faceted, etc.)
Length and breadth of knapping surface
Scar pattern of the last negatives on the knapping surface of the core
Presence of conchoidal negatives (yes/no)
Presence of fissuration in overhang or striking platform (yes/no)
Presence of bluntness in overhang or striking platform (yes/no)
Freehand or bipolar core
Type of quartz core following Fig 4

**Table 3:** Variables recorded for cores (non-quartz rock types, such as hornfels, dolerite, sandstone, etc.).

Type of blank
Presence of cortex (0, 1–10, 10–40, 40–60,60–90, 90–99, 100%)
Length, breadth, thickness (maximal measures for the three of them) and weight
Volumetric shape (prismatic, conical, globular)
Lateral trimming (crest or semicrest) (yes/no)
Number of lateral trimmings
Other type of trimming
Number of striking platforms
Orientation of the striking platforms
Preparation of the striking platforms (simple flake, faceted, etc.)
Shape of the striking platforms
Shape of the exploitation surface
Length and breadth of the striking platform (maximum measurements)
Length and breadth of the exploitation surface (maximum measurements)
Curvature of the exploitation surface (yes/no)
Length and breadth of last negative (not taking into account accidents)
Angle between striking platform and exploitation surface for prismatic core, or angle between faces for discoidal and Levallois cores in the 4 subdivisions.
Type of core: prismatic, discoidal, Levallois, Howiesons Poort core, Core on flake (bipolar core, *Kostienki* core, burin-like core, end scraper core). These general categories were previously used and explained in [36].

**Table 4:** Variables recorded for all unretouched blanks.

Type of blank (flake, bipolar blank, burin spall)
Length, breadth, thickness (maximum measurements for the three of them)
Presence of cortex (0, 1–10, 10–40, 40–60,60–90, 90–99, 100%).
Type of platform: plain, cortical, dihedral, faceted, punctiform, linear. Removed, broken/crushed.
Length and breadth of the platform
Angle between platform and dorsal face of the flake
Number of scars on the dorsal face
Dorsal scar pattern: unidirectional, unidirectional convergent, bidirectional, crossed, subcentripetal, centripetal and unknown.
Shape of the blank/ lateral edge: Converging, expanding, ovoid, circular, indeterminate.
Cross section: triangular, right-triangular, trapezoidal, lenticular, domed, indeterminate.
Accidents (yes/no)
Type of accident (overshoot, hinge/step)

**Table 5:** Variables recorded for retouched blanks.

All the attributes previously listed for blanks without retouch in Table 4.
Location of retouch
Delineation of the edge/s retouched (rectilinear, curved, denticulated)
Regularity of the retouch (yes/no).
Continuity (yes/no)
Type of morphotype including: Notch, end-scraper, side-scraper, strangulated piece, denticulate, burin, marginal retouch flake/blade, retouch flake/blade, borer, bifacial piece and backed piece (including all the categories illustrated in Fig 5). All these morphotypes were already recognised in the GS study of Sibudu [36]

The statistical analyses have two main objectives: (1) to describe better the studied assemblage and, (2), to identify and explore relationships between the different recorded quantitative and qualitative variables. The null hypothesis was that all rock types were knapped in the same manner. In other words, all types of rocks were reduced following identical knapping methods; and therefore an identical size distribution of blanks can be found for each one of the rock types. In order to examine the variability of the different recorded variables, a number of descriptive means was employed such as histograms, dispersion diagrams, box-plots, univariate statistics, etc. For the relationships among the different quantitative and qualitative variables recorded in the attribute analysis I performed different basic statistical analyses. Shapiro-Wilk normality tests were performed to identify those quantitative variables that had normally distributed values. Additionally, mixture analyses for the non-normal distributions of the quantitative data were implemented, in order to find potential groups of quantitative data with normal distributions [74]. As explained by Soto Sebastián [74]:158 mixture analysis is an agglomerative, hierarchical and univariate statistical test. It is a method of maximum likelihood estimation to recognize parameters (such as means and standard deviation) of two or more univariate normal distributions grouped in a single sample. For example, in order to discover whether, within a blank category such as blades, different knapping methods were applied, different normal groups of blanks would be expected (see an application of this methodology in lithic analysis in Rios et al. [75] and Soto Sebastián [74]. Besides, t-tests (for normal distributions) and U Mann-Whitney (for non-parametric distributions) were completed for comparisons of quantitative sets of data. Finally, chi-square tests were applied to search for relationships amongst qualitative data (for example, between cortex distribution and type of blank or between shape of the blank and the dorsal scar ordinations). All these analyses were performed with the open-access software package PAST (http://folk.uio.no/ohammer/past/).

## Results of the Grey Rocky Lithic Industry Technological Analysis

### The Cores

#### What type of knapping methods can be inferred from the technological analysis of the cores?

**Fig 6:**
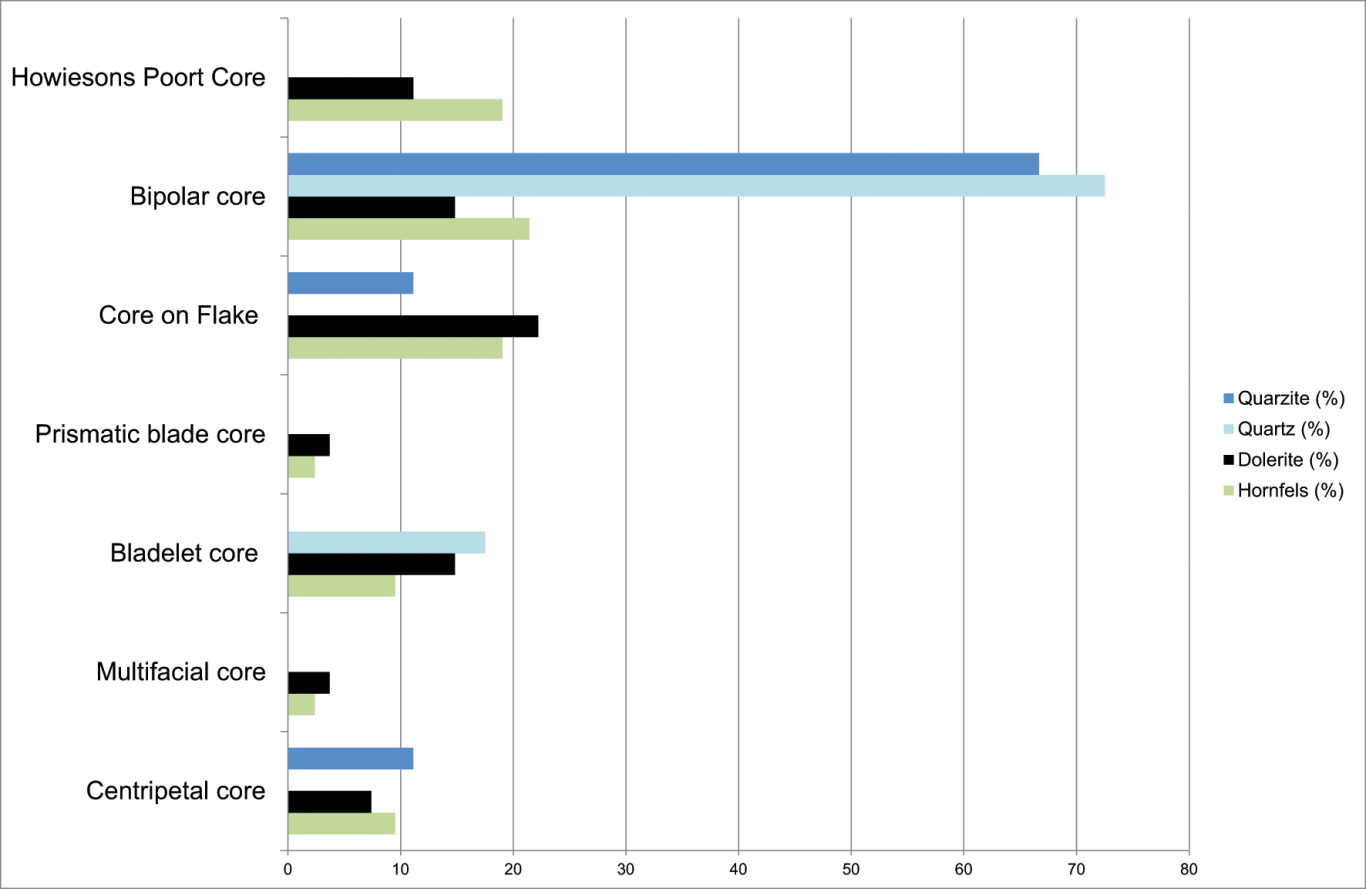
Grey Rocky core types by raw material type.

The first striking characteristic of the cores is that those made from hornfels and dolerite are quite similar. A variety of different cores types, such as core on flakes, Howiesons Poort cores, bladelet cores, centripetal cores, etc. were made from these materials, and they are very different from cores made on other rock types such as quartz or quartzite, for which the majority of cores is bipolar(Tables 6 and 7; and Fig 6). Therefore, the percentages of core types and their qualitative characteristics imply that these different broad classes of raw material were managed and reduced differently.

**Table 6:** Grey Rocky core types by raw material type. *See quartz subtypes cores in Table 7.

CORES GR	Hornfels (N)	Hornfels (%)	Dolerite (N)	Dolerite (%)	Quartz (N)	Quartz (%)	Quarzite (N)	Quarzite (%)	Sandstone (N)	Cryptocristalline (N)
**Centripetal core**	4	9.5	2	7.4	0	0	1	11.1	0	1
**Multifacial core**	1	2.4	1	3.7	0	0	0	0.0	0	0
**Bladelet core (from a small nodule)**	4	9.5	4	14.8	7	17.5	0	0.0	0	0
**Prismatic blade core**	1	2.4	1	3.7	0	0	0	0.0	0	0
**Core on Flake (burin-like, end-scraper-like, *Kostienki*)**	8	19.0	6	22.2	0	0	1	11.1	0	0
**Bipolar core**	9	21.4	4	14.8	29	72.5	6	66.7	1	0
**Howiesons Poort Core**	8	19.0	3	11.1	0	0	0	0.0	0	0
**Indeterminate**	7	16.7	6	22.2	4	10	1	11.1	0	0
**TOTAL**	**42**	100	**27**	100.0	**40**	100	**9**	100	**1**	**1**

**Table 7:** Type of cores in quartz (the types, such as B1 and B2) are illustrated in Fig 4. Ind = indeterminate.

Type	N
**B1**	2
**B2**	22
**B3**	2
**B4**	3
**F10**	3
**F2**	2
**F3**	1
**F9**	1
**Ind**	4
**TOTAL**	**40**

**Fig 7:**
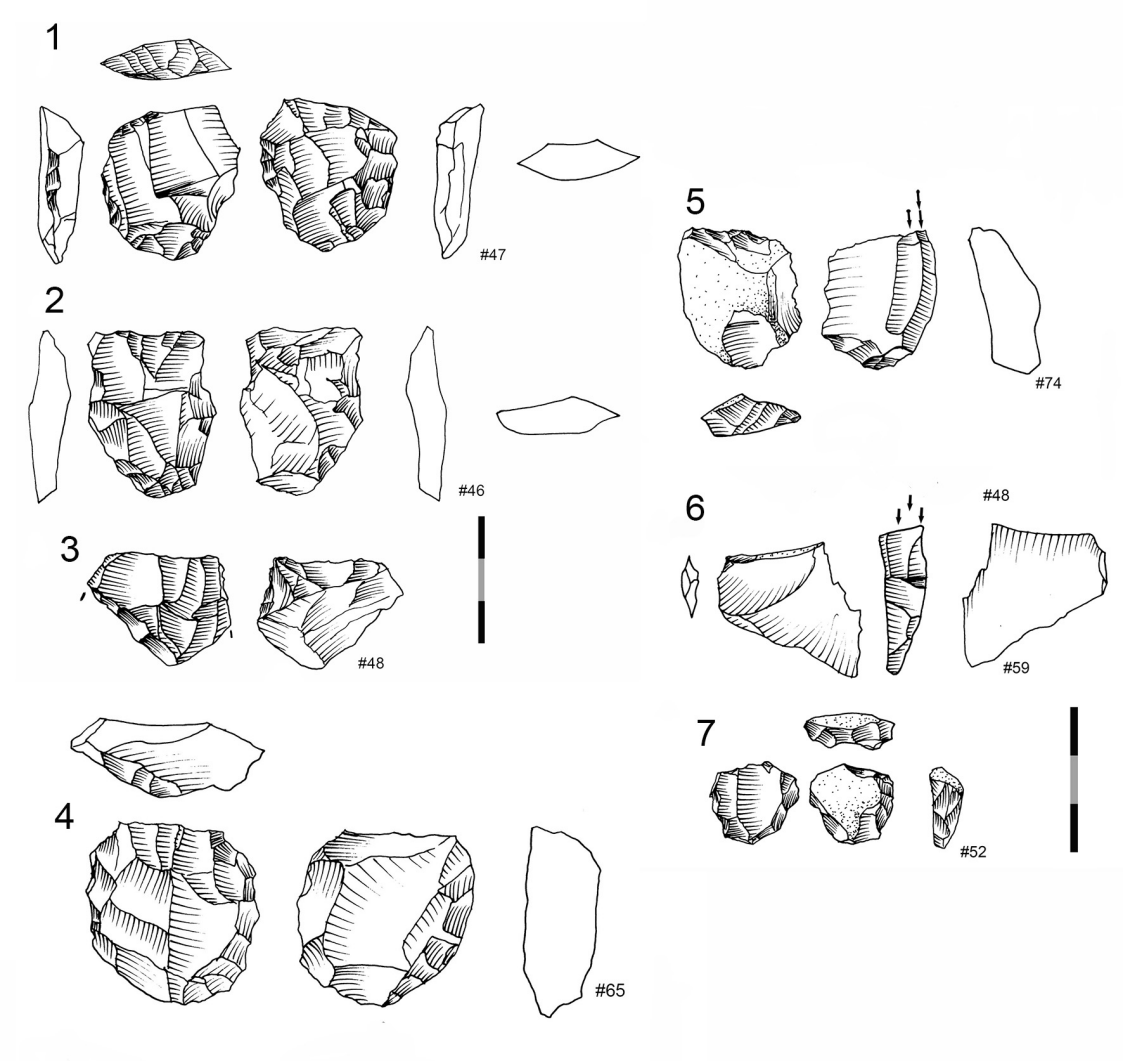
Dolerite and hornfels cores from GR layer. 1 Core on flake (*Kostienki*), hornfels. 2 and 3 HP core, hornfels. 4. HP core, dolerite. 5. Core on flake (burin core), dolerite. 6. Core on flake (burin core), hornfels. 7. Bladelet core on small nodule, hornfels.

Another general characteristic is that the percentages of cores by rock types differ from those of rock types represented in the rest of the lithic assemblage recovered from GR. Hornfels cores are more abundant than those made from dolerite, and quartz more than sandstone (*cf*. Fig 3, Table 6 and Fig 6). One must keep in mind, though, that these patterns may be due to sampling since Wadley’s excavations were from only a small portion of the site’s archaeological deposits (6m^2^). Most of the hornfels and dolerite cores are core on flakes, bipolar cores (which usually are made also on flakes) and the so-called Howiesons Poort Cores [25, 34] (Table 6). Within the category core on flakes there are different sub-types such as *Kostienki* cores or burin-like cores (Fig 7). These sub-types are not a novelty in the HP because they were already recognized in the older GS layer of Sibudu [36]. These cores on flakes and the bipolar cores were most likely geared towards the production of bladelet blanks.

The Howiesons Poort Cores (HP cores) have a similar pattern to those described in Wurz [25], Villa et al. [34] and in the Sibudu GS analysis [36]. The striking platform of a HP core is prepared by creating a large truncation (or faceted surface) and the lateral convexities are produced via semicrest removals. Sometimes the distal part of the knapping surface has small removals to give the core morphology a narrow end. The other face of the core (the non-knapping surface) is usually prepared with centripetal removals. These HP cores were also probably intended for blade/bladelet production. As mentioned in de la Peña and Wadley’s [36] discussion of Sibudu’s GS layer, some cores on flakes follow this HP core-morphological pattern and these types are recognized in the Paleolithic literature as *Nahr Ibrahim* or *Kostienki* cores [76–80](see Fig 7 #1).

**Fig 8:**
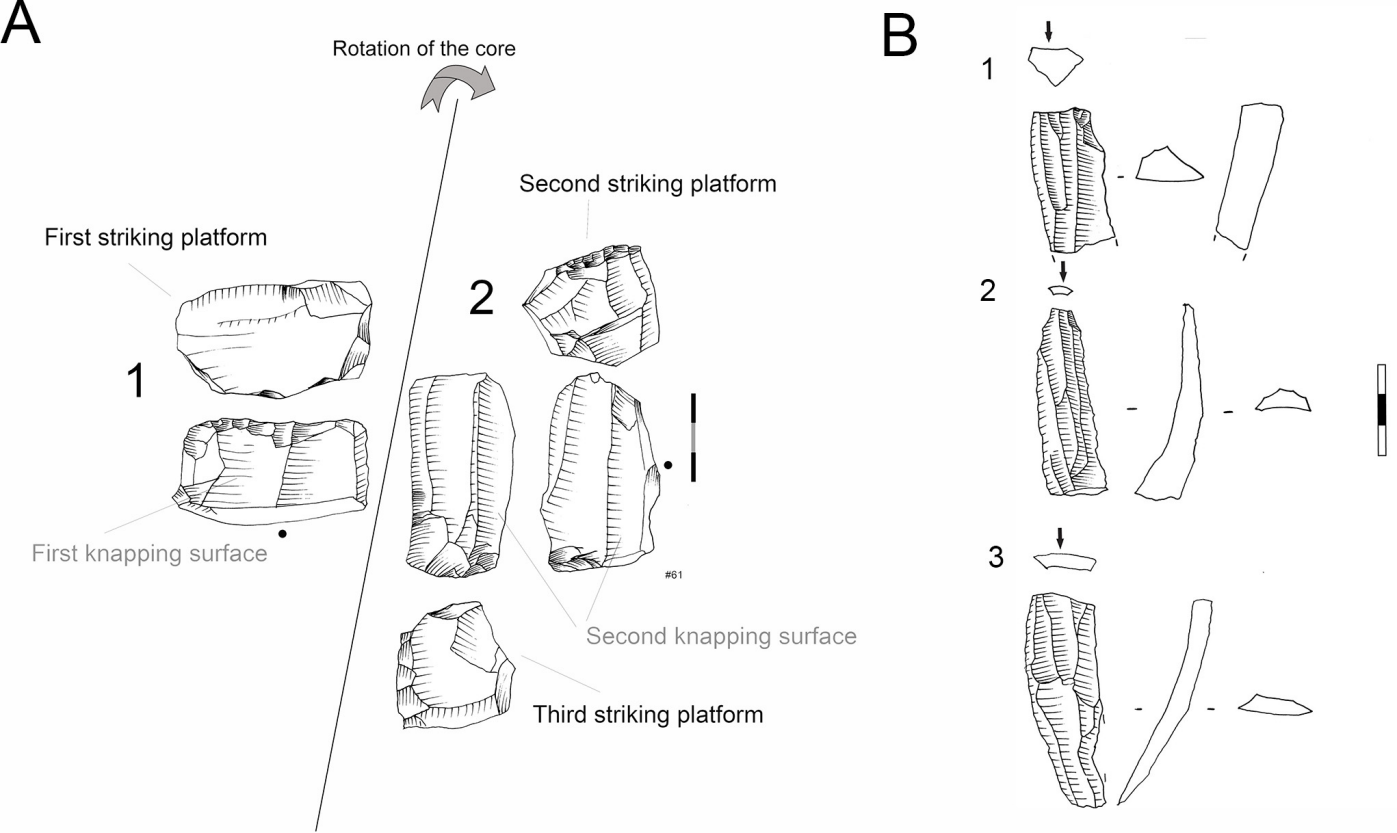
Prismatic blade core example in GR. A. Prismatic blade core (#61) (dolerite) showing a change of direction in the knapping. 1. First striking platform and exploitation surface. Afterwards the core was rotated in order to continue the blade production. 2. Second and third striking platform (opposed) and knapping surface. B. Three dolerite blade blanks that could correspond to a prismatic core as example #61. 2. Is an overshoot.

Blade prismatic production is revealed by one dolerite core (Fig 8) and one hornfels core. The dolerite example exhibits a change of knapping direction during the reduction sequence, which is a characteristic that is evident elsewhere in the high percentage of false semi-crests in this material type, as well as with hornfels (*vid*. *infra*).

In addition, there are multifacial cores and cores with centripetal scar patterns (Table 6). However, none of these examples falls within the *Levallois* technological definition [81–83]. In other words, they have a centripetal scar pattern, but they do not have two hierarchical surfaces characteristic of *Levallois* cores. Importantly, these cores demonstrate that there was an interest in the production of flakes from hornfels and dolerite.

The percentages of cores on hornfels and dolerite (with a notable representation of cores on flakes) suggest that there was a great emphasis on bladelet and small flake production. This would, however, be a biased conclusion because the study of the non-retouched blanks (*vid*. *infra*) reveals that the knapping strategies were very broad, and did not only focus on small blanks.

**Fig 9:**
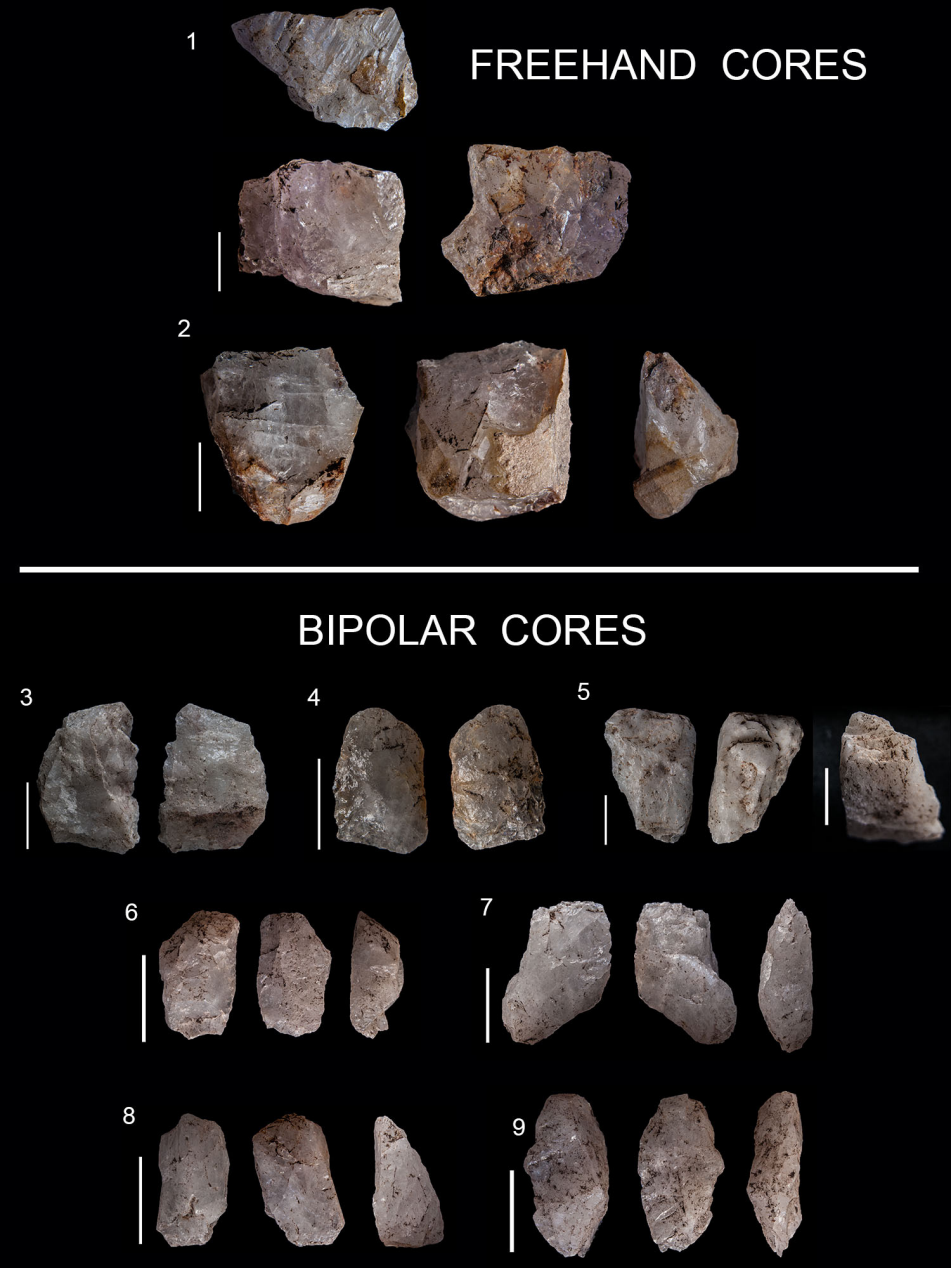
Freehand and bipolar quartz cores in Sibudu, layer GR. Scale 1 cm.

**Fig 10:**
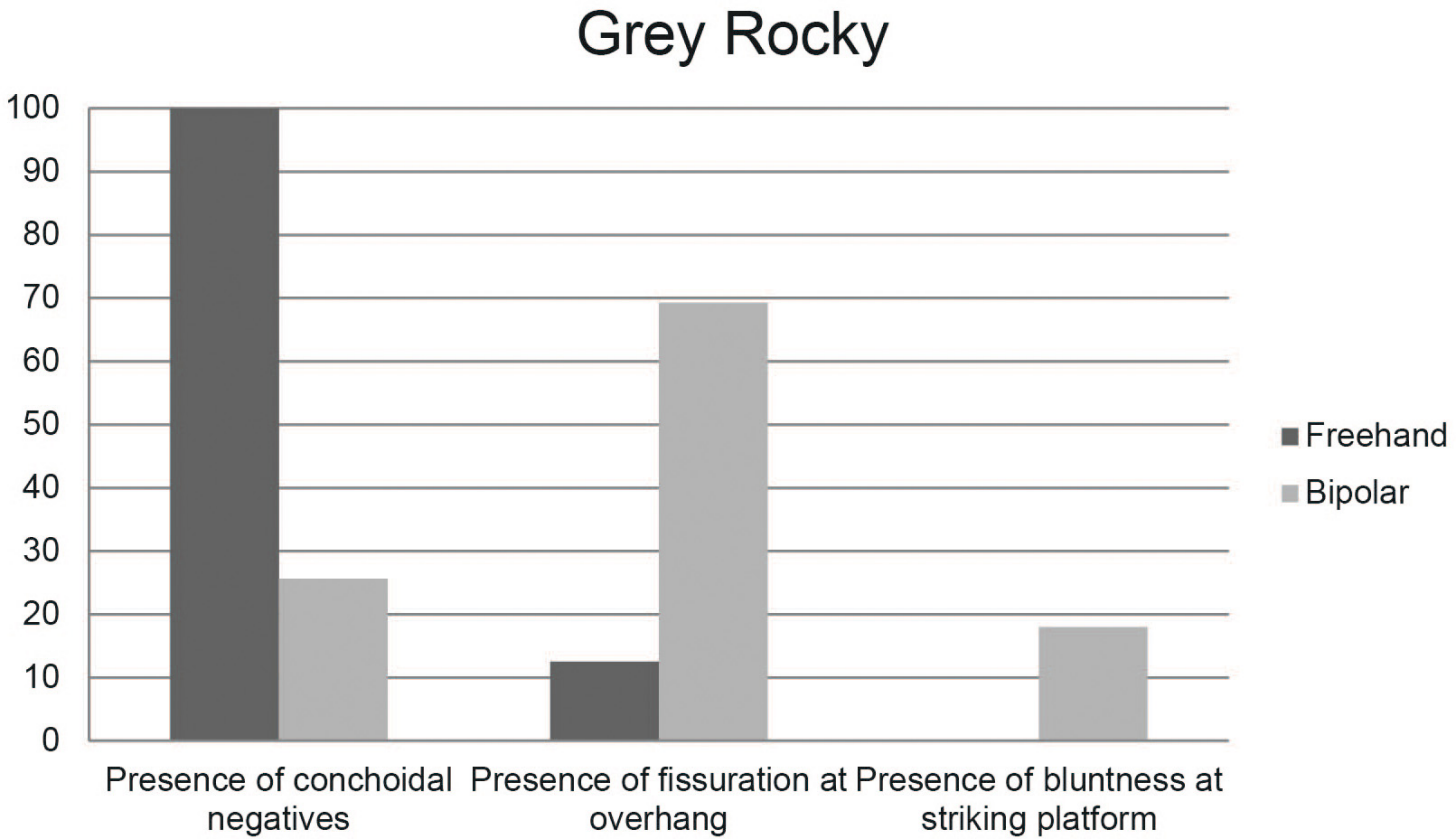
Percentage of conchoidal negatives, fissuration and bluntness of freehand and bipolar cores in Sibudu, layer GR.

Quartz cores are divided into two main categories: freehand bladelet prismatic cores and bipolar cores (Fig 4). The different subtypes considered in this study (Fig 4) are presented in Table 7. It seems that the freehand bladelet cores were recycled into bipolar cores, as was the case for quartz management in the GS layer [37]. Indeed, the percentage representation of freehand and bipolar cores in GR is similar to that in the other HP layers (GS and PGS) see [84] and Fig 4 within it. The distinction between freehand and bipolar cores is clear from a qualitative point of view (see in this regard Fig 9). Bipolar cores have abundant fissuration at the overhang, and bluntness of the striking platform, whereas conchoidal scars are virtually absent [37, 84]. On the contrary, freehand quartz cores have the opposite tendency (see Fig 10 for the percentage of these qualitative characteristics). The recycling of freehand cores into bipolar cores is also supported by the representation of cortex in these two categories, see [84] and Fig 8 within it. I have not used statistical analysis to distinguish between these two types of cores because of the small number of freehand quartz cores (n = 9) in GR. In the case of quartz, freehand prismatic bladelet cores and bipolar cores explain most of the quartz non-retouched blanks. However, as demonstrated in de la Peña et al. [41], there is also quartz bifacial point production in GR, and these points were made on flakes. However, there are no discoidal or centripetal cores for obtaining blanks for the quartz points.

**Fig 11:**
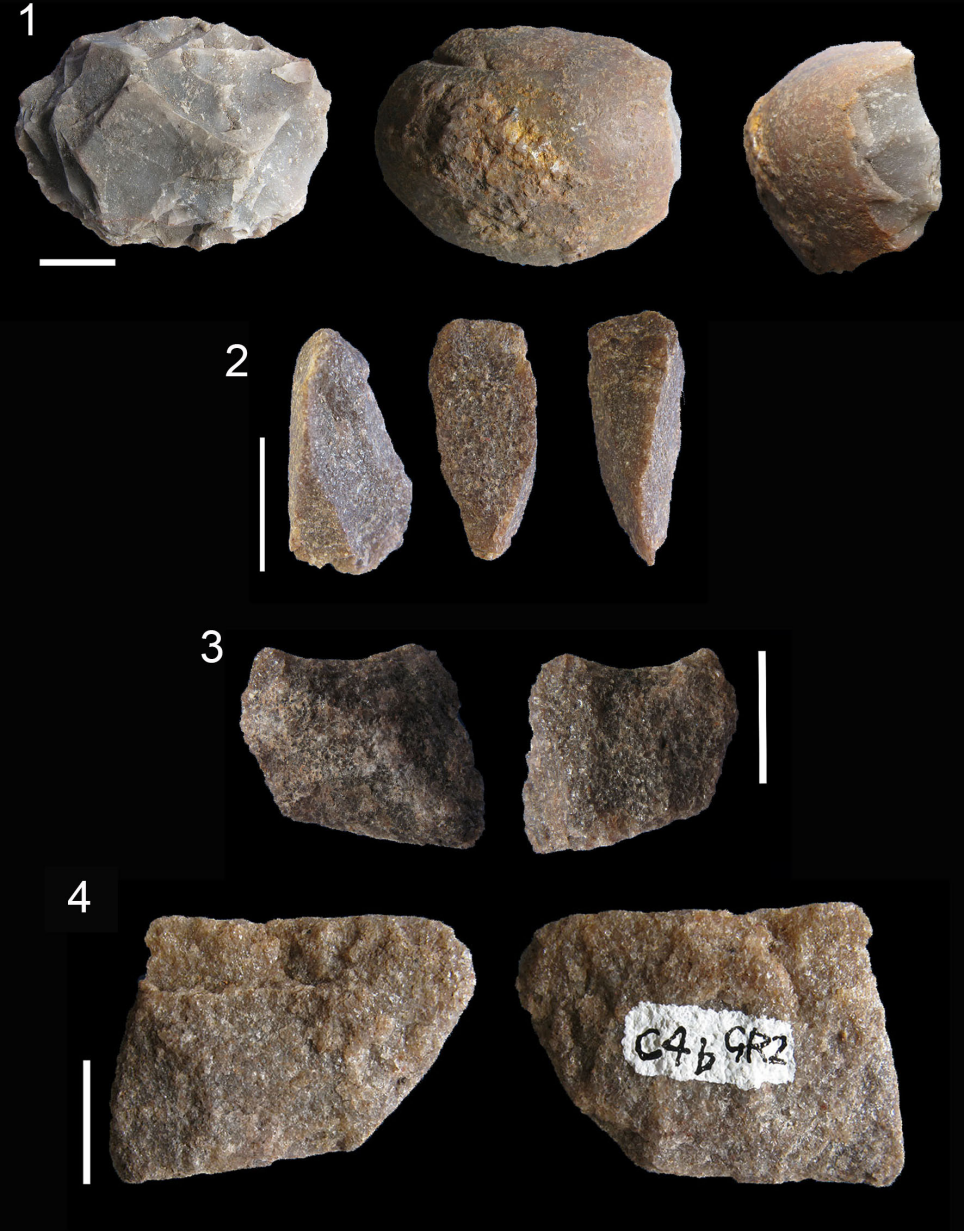
Examples of CCS and quartzite cores in Sibudu, layer GR. 1. Centripetal core (*Levallois*-like) on CCS. 2, 3 and 4. Bipolar quartzite cores. Scale 1 cm.

There is an exhausted centripetal core (Fig 11 #1) made from a river pebble of CCS. There are also some bipolar quartzite cores (Fig 11 # 2, 3 and 4) and one centripetal quartzite core. The sandstone cores do not give us many clues about the management of this rock type because there is only one sandstone blade prismatic core and it is difficult to read owing to the characteristics of this rock type.

#### What the typometric analysis of the cores shows

**Fig 12:**
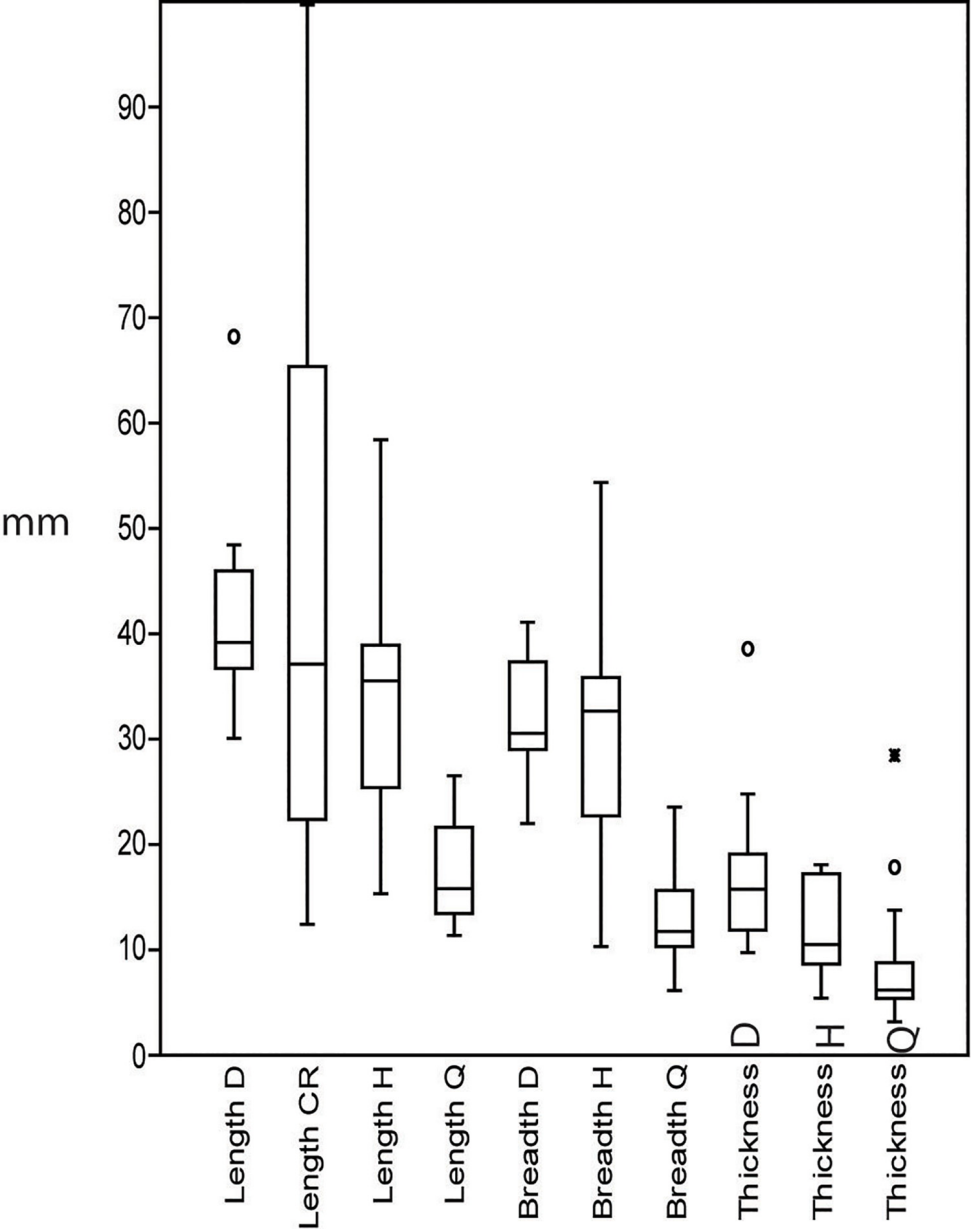
Box-plot of the length, breadth and thickness of dolerite, hornfels and quartz cores. Besides dolerite length there is ‘Length CR’, which is the box-plot of dolerite crest and semicrest and false semicrest, as it can give also an idea of the length of the cores. As can be seen, the measurements are clearly different among rock types, only breadth of dolerite and hornfels show some overlap.

The typometry of the cores shows that there is a clear difference in length, breadth and thickness for three of the rock types: dolerite, hornfels and quartz (Fig 12). This difference is especially acute in the case of quartz, where the majority of pieces is less than 2 cm in length because of the final bipolar reduction of these cores, which supports a truly microlithic strategy of recycling for the production of small blanks.

An ANOVA analysis verified this intuitive perception for the comparison of length, breadth and thickness of these three rock types [p (same) for Length: 1.215 E-13; Breadth 5.467 E-15; Thickness: 5.005E-6]. Moreover, the proof of ‘honestly significant difference of Tukey’ also gave us noteworthy differences, particularly for length, where the differences were significant between each of the three rock types.

### The Core Related By-Products

**Fig 13:**
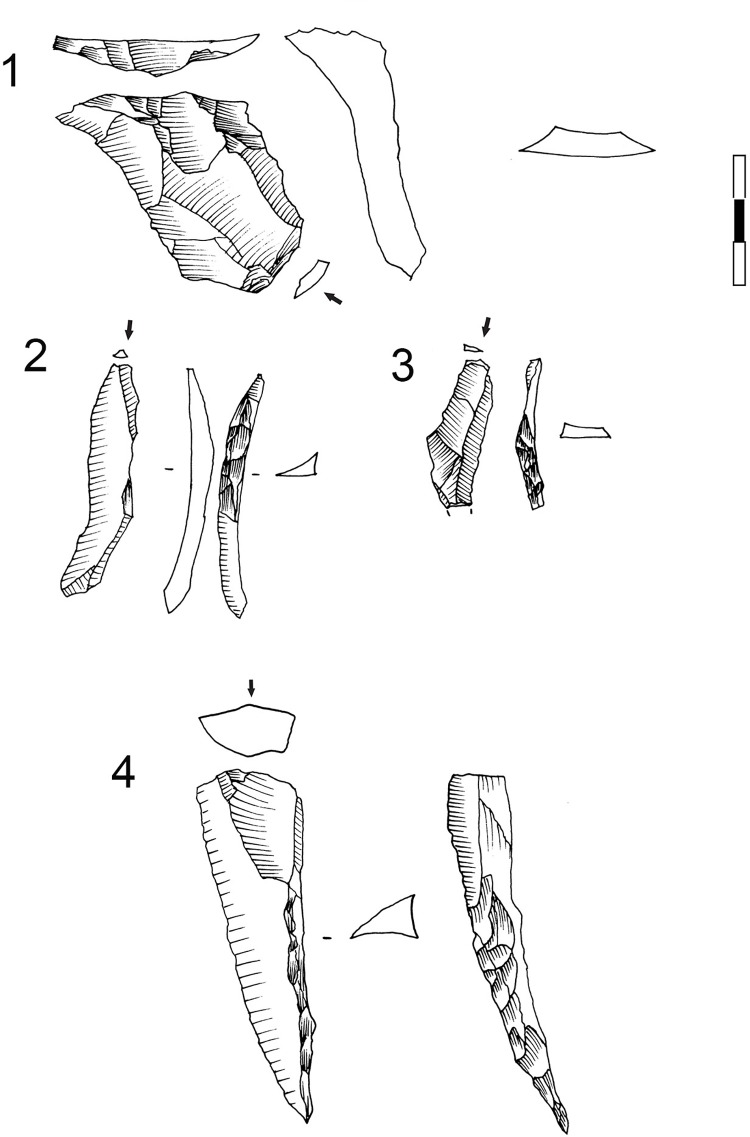
Core related by-products examples in Sibudu, layer GR. 1. Overshoot hornfels flake showing the morphology of a prismatic core with two opposed striking platforms. 2 and 3. Semicrest in hornfels. 4. Semicrest in dolerite from a big prismatic blade core. Scale 3 cm.

**Fig 14:**
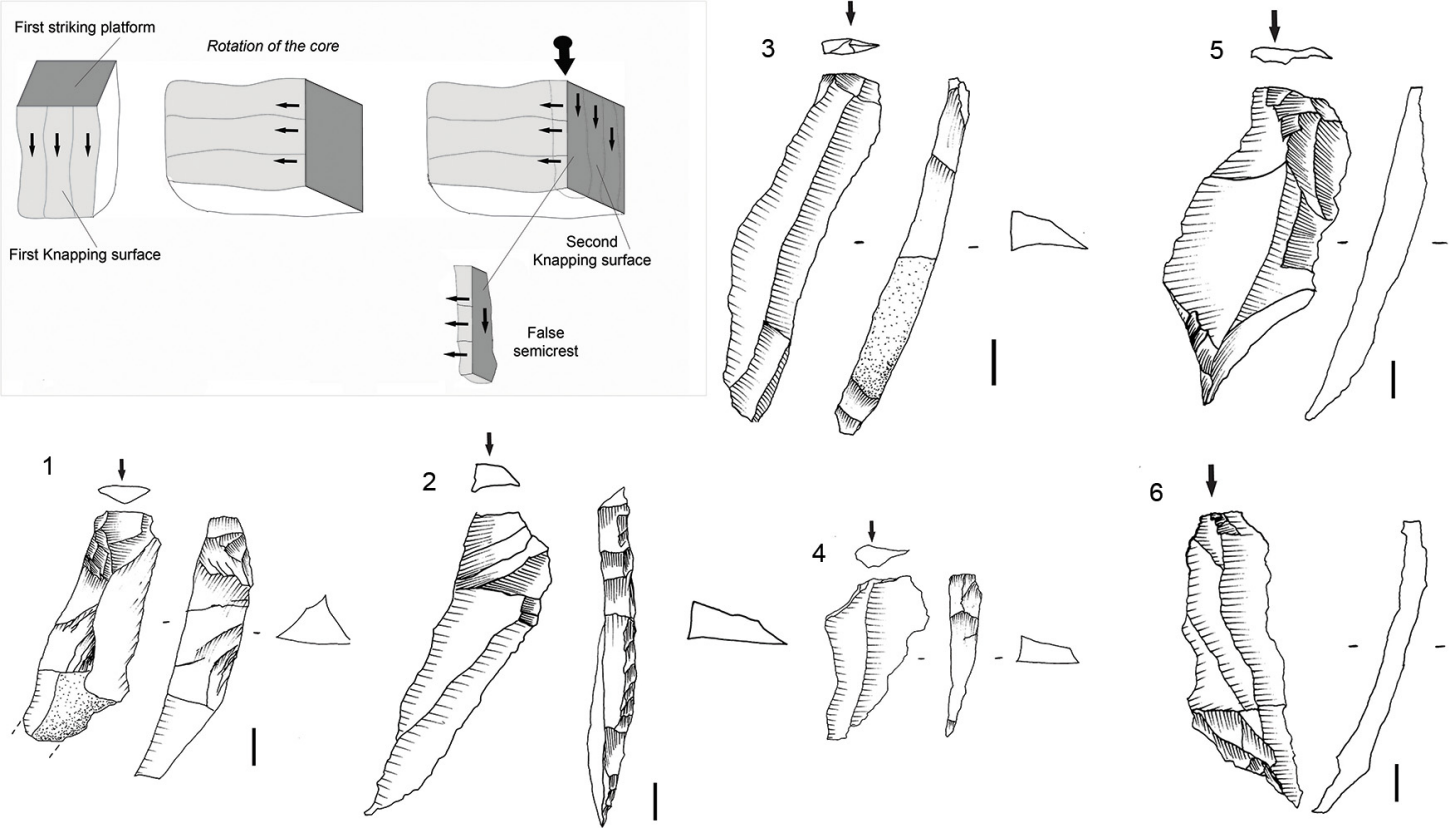
Core related by-products-False semicrest examples in Sibudu, layer Gr. These blanks are the result from a change in the direction of blank removal with the crest representing a previous overhang. On the top left schematic drawing explaining the production of these types of blanks. 1, 2, 3 and 4 dolerite examples. 1, 2 and 3 are coming from big blade prismatic cores as the one shown in Fig 8. 5 and 6. Hornfels examples. Scale 1 cm.

The core related by-products that I separate in this study are only in hornfels and dolerite (Figs 13 and 14). For the rest of the rock types I did not find any blank that could be included within my definition of core-related by-products as given in the *Methodology* section. As can be seen in Table 8, most of the core related by-products in hornfels and dolerite fall within two broad categories. On the one hand, many of them were produced to start or correct blade prismatic cores (crest and semicrests) (Fig 13). On the other hand, there are several blanks which denote a change of direction in the reduction sequence of blade prismatic cores; this is what I have decided to call ‘false crests and semicrests’ (see Methodology section) (Fig 14). These by-products of the blade prismatic reduction sequence might signify that big prismatic blade cores were heavily reduced by a change of knapping direction during the production of blades (as shown by the dolerite big blade prismatic core of Fig 8).

**Table 8:** Numbers and percentages of core related by-products in GR for dolerite and hornfels.

Core related by-products in GR	Dolerite	%	Hornfels	%
**Crest**	2	4.55	0	0
**Semicrest**	7	15.9	2	10.53
**Cleaning flakes**	5	11.4	0	0
**False semicrest**	20	45.5	13	68.42
**Flake showing core morphology**	7	15.9	2	10.53
**Knapping accidents**	3	6.82	2	10.53
**TOTAL**	**44**	**100**	**19**	**100**

### The Blanks without Retouch

#### Do the non-retouched blanks show evidence for other knapping methods?

The complete blanks are mainly dolerite, hornfels and sandstone flakes (see S1 File). These are the three main rock types in this layer (Fig 3).

**Fig 15:**
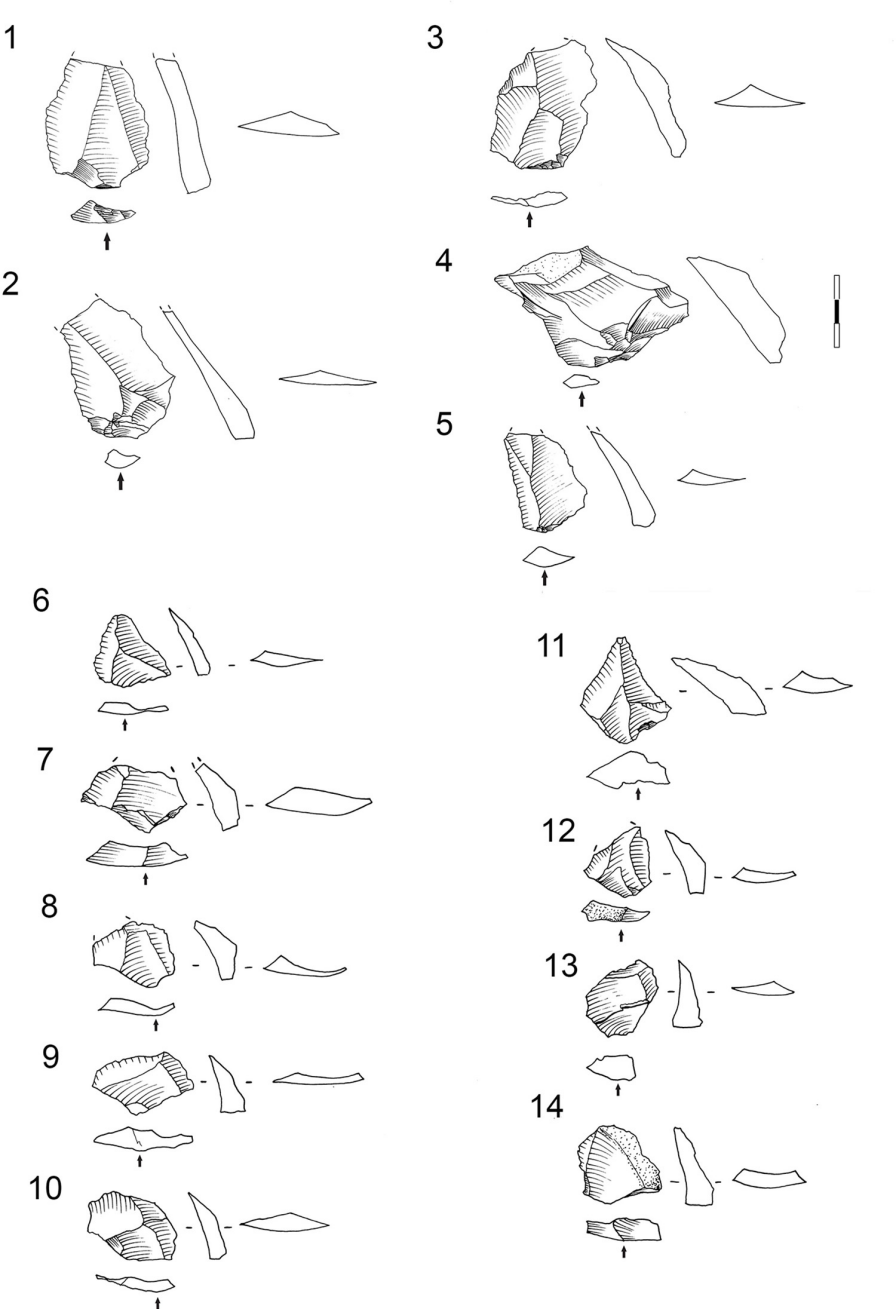
Different examples of dolerite flakes in Sibudu, layer GR. 1 to 4 seems related to a *Levallois* reduction sequence, whereas pieces such as 6 or 11 could be related to a discoidal knapping method or a preparatory phase of a *Levallois* core. Scale 3 cm.

During the analysis typical by-products of *Levallois* production have been observed for dolerite. This situation is quite different from what was seen during the core analysis. Some of the *Levallois* flakes in this assemblage follow the canonical typological description of these type of blanks: symmetrical flakes with centripetal scar ordinations (more than three negatives), longer that wider, and with rectangular or quadrangular shapes [85]. However, the platforms are not always faceted, and plain and dihedral platforms are abundant. For dolerite there are also some flake by-products that could correspond to a discoidal knapping method, but they could also come from a preparatory phase of a *Levallois* core (Fig 15). As other researchers have pointed out [86], it is not an easy task to detect *Levallois* reduction only from the blank production. Furthermore, making a distinction between discoidal and *Levallois* based on blank production is particularly problematic. Indeed, the topic has been intensely debated in Palaeolithic literature for some years [87]. Nonetheless, what it is interesting is that it is clear that for dolerite the production of flakes was intentional and was not only a subsidiary of blade production. The type of dolerite blanks illustrated in Fig 15 #1 to 4, seem clearly related to a *Levallois* flake reduction sequence. In addition, some of the dolerite flakes originate from the preparation of blade cores.

In hornfels, on the contrary, I did not find any typical *Levallois* flakes. In addition, most of the completed blanks without retouch seem associated with blade/bladelet production. However, it seems that there was also deliberate production of hornfels flakes, as is already demonstrated from the centripetal and multifacial cores.

Moreover, for dolerite it is clear that big prismatic blades with unidirectional or bidirectional scar patterns and triangular or trapezoidal sections (Fig 8B) probably come from prismatic cores like the one shown in Fig 8A. The same type of big prismatic blade also occurs on hornfels. Moreover, the cores and the core-related by-products confirm this type of reduction by the presence of many false semicrests (Fig 14). Therefore, the information from the blanks without retouch supports that from the cores and the core related by-products. However, the abundance of these big prismatic blades seems very important judging from the number of them, whereas in the core sample they were weakly represented (only one prismatic blade core for hornfels and one for dolerite).

In regard to the blade/bladelet production for hornfels and dolerite, it must be stressed that probably the thin blades with slight curvatures in the distal part of their profiles were produced from HP cores and not from prismatic cores (but experiments should be performed in order to confirm this suggestion).

Sandstone is the third rock type represented in the flake study and it also reflects a significant centripetal flake production and a big blade production.

All the previous remarks were made from the identification of typical flakes and from the shape of the blanks (see S1 File). Therefore, it could be argued that the study was made subjectively. In order to give a more objective perspective on the strategies of knapping it would be appropriate to record the frequencies of various qualitative attributes. Moreover, this type of approach can give us a better idea of the representation of the different knapping methods previously documented qualitatively for each of the rock types.

**Fig 16:**
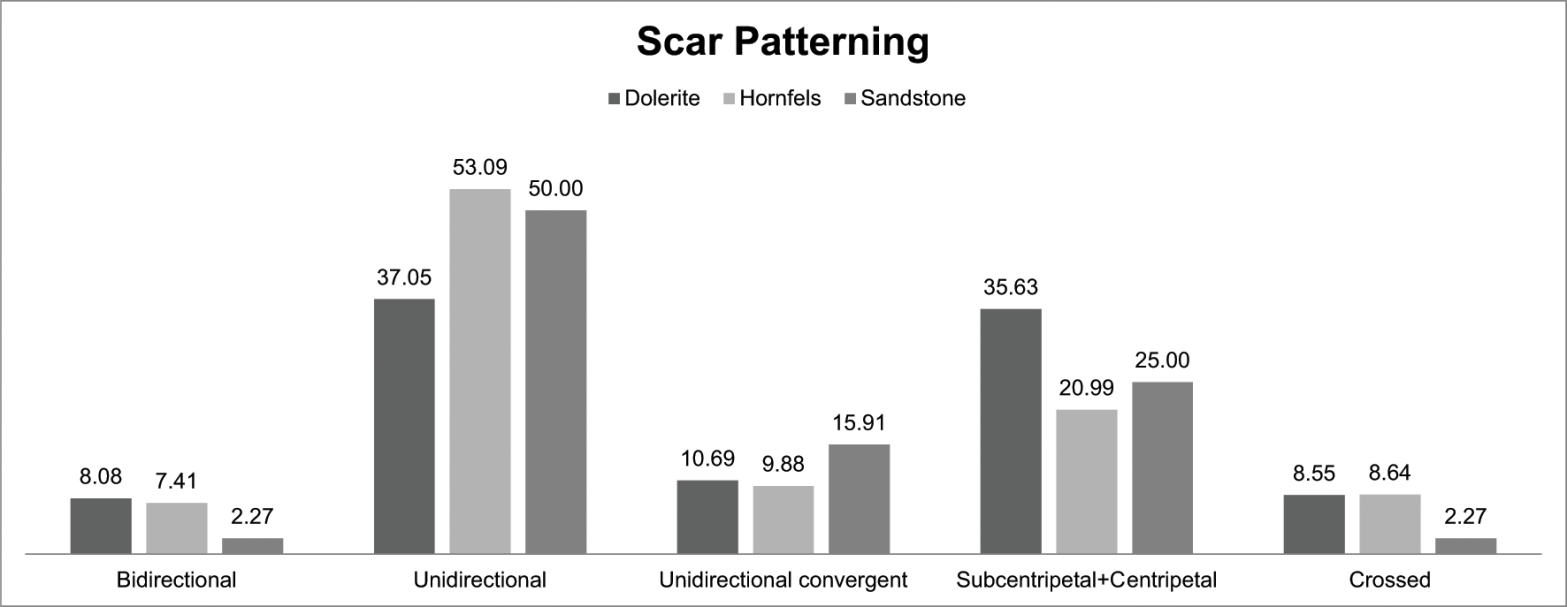
Flake scar patterning for dolerite, hornfels and sandstone in Sibudu, GR layer.

It is interesting to see the scar pattern distribution for the different rock types. As can be seen in Fig 16, dolerite has a high percentage of centripetal and subcentripetal scar patterns (~36%), which makes sense with *Levallois* or discoidal strategies. The unidirectional scar pattern is also important (~37%), and it mainly corresponds with the blade knapping methods that characterize some of the blanks and the cores (prismatic blade production, HP cores and core on flakes). In contrast, the percentage of unidirectional scars on hornfels and sandstone is higher than for dolerite, which might indicate that for these two rock types blade/bladelet knapping methods were more common (~50/53%) than flaking methods.

**Fig 17:**
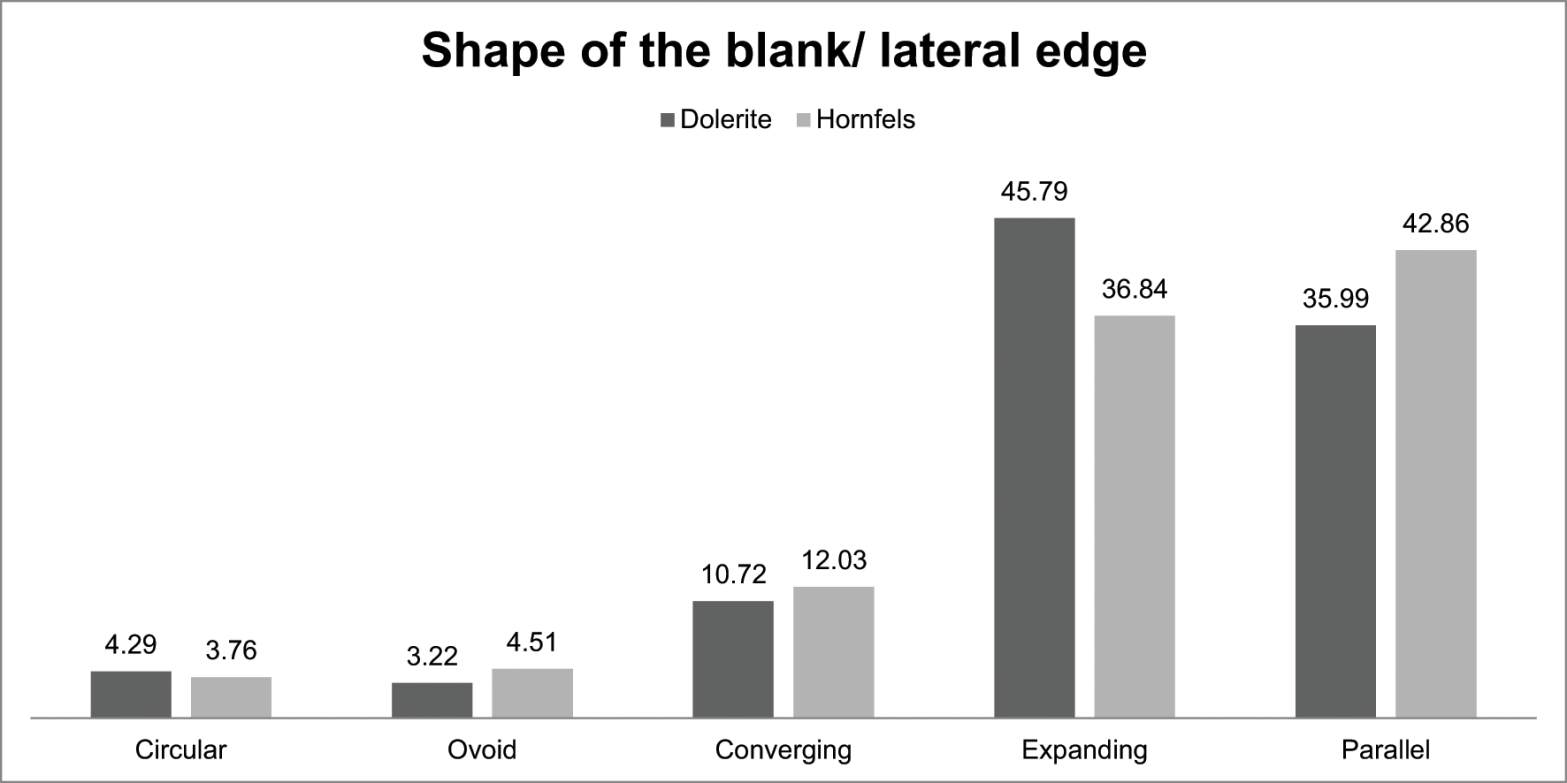
Lateral edge for dolerite, hornfels and sandstone in Sibudu, layer GR.

**Fig 18:**
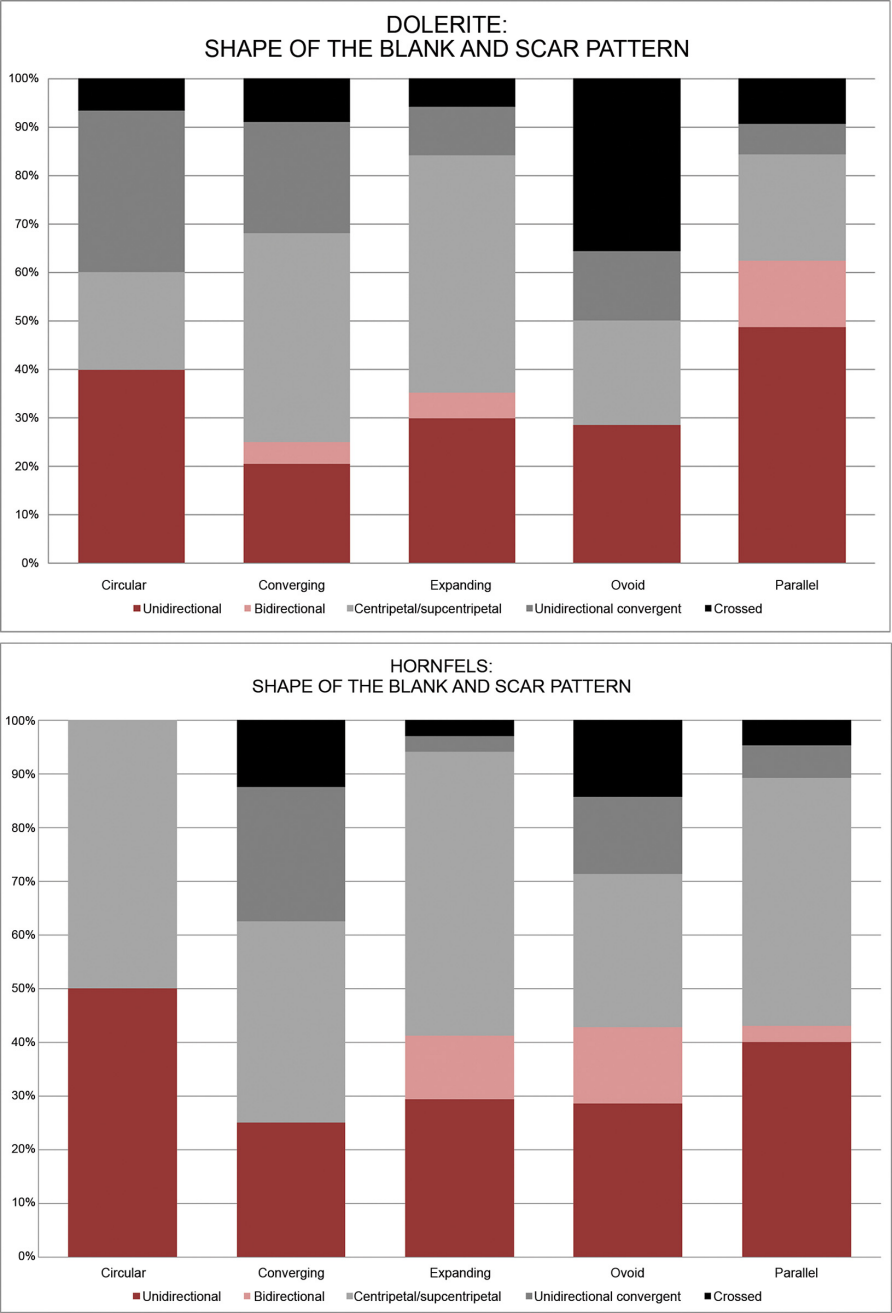
Dorsal scar pattern and shape of the blank for hornfels and dolerite in Sibudu, layer GR.

This same line of reasoning can be applied if we look at the shape of the blanks. On the one hand, as can be seen in Fig 17, dolerite has a higher percentage of expanding shape blanks than the other rock types. On the other hand, hornfels and sandstone have a higher percentage of parallel-edged blanks than dolerite; this would also support the idea that flaking methods (such as *Levalloi*s, discoidal or multifacial) were favoured more often for dolerite than for hornfels and sandstone in this layer. These interpretations are supported by Fig 18 which illustrates dorsal scar pattern and shape of the blank for hornfels and dolerite. As can be seen, dolerite flakes with expanding and converging edges (which are consistent with flake production) have a higher percentage of centripetal/subcentripetal and unidirectional convergent scar patterns. The same logic applies to hornfels where converging and expanding flake shapes have a notable representation of unidirectional convergent and centripetal/subcentripetal scar patterns.

Furthermore, the Chi^2 analysis of the scar pattern and shape of the blank in the three main rock types demonstrates that the different categories have remarkably different groupings. This might imply different manufacturing strategies for hornfels and dolerite (sandstone frequencies were too small to perform the Chi-square test) [Scar Pattern: Dolerite: Deg. freedom:4, Chi^2: 188.42, p(same):1.1594E-39. Hornfels: Deg. freedom:4, Chi^2:60.173, p(same):2.6681E-12. Shape of the blank: Dolerite: Deg. freedom:4, Chi^2:501.3, p(same): 3.5144E-107. Hornfels: Deg. freedom: 4; Chi^2: 91.323, p(same):6.8922E-19].

**Fig 19:**
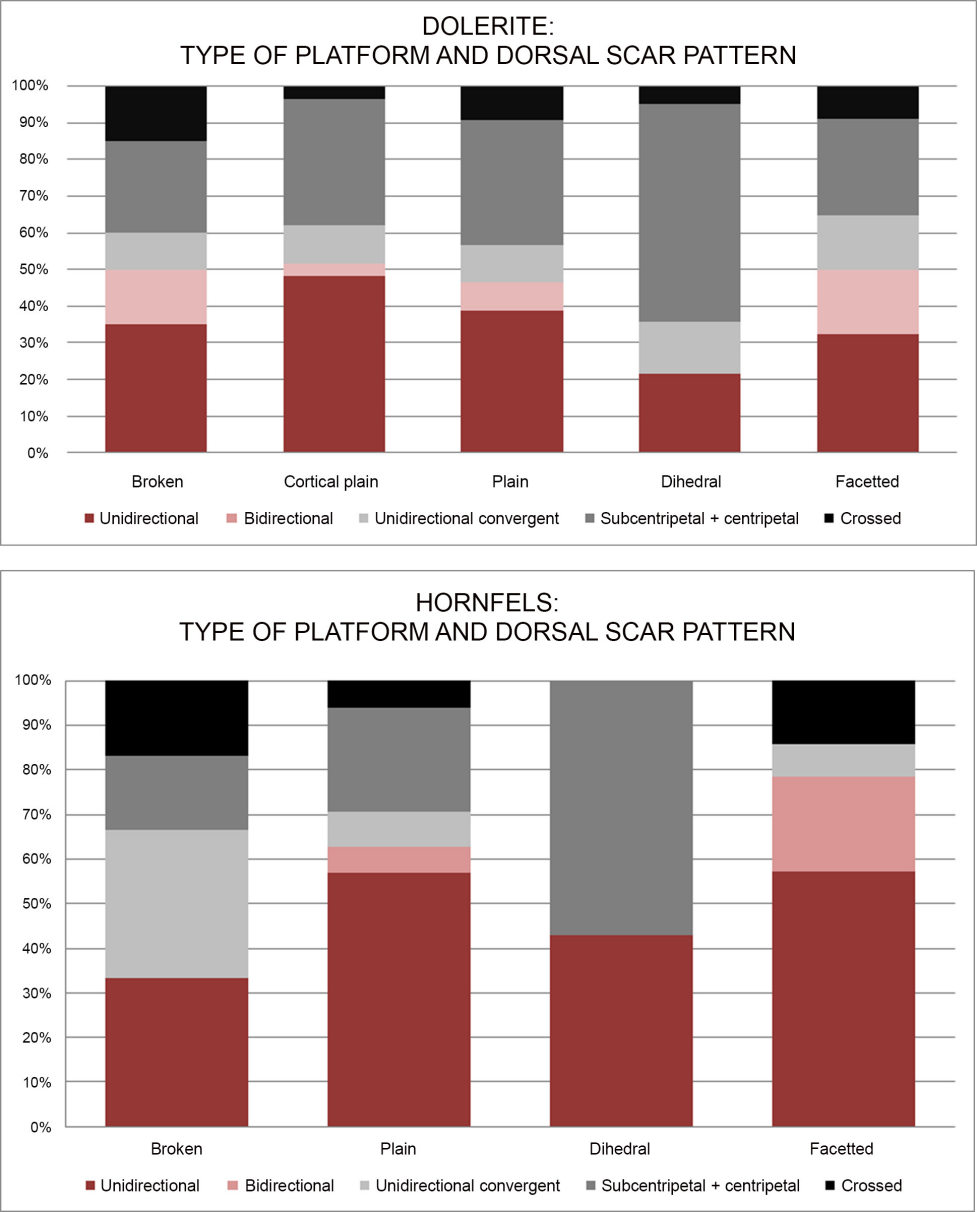
Type of platform and dorsal scar pattern for hornfels and dolerite in Sibudu, layer GR.

The frequencies of platform types give clues to the trends of knapping for the different rock types. For example, hornfels has 18.35% of faceted platforms, probably related to the blade/bladelet production. Dolerite has a higher percentage of dihedral production, which I emphasized earlier. Finally, sandstone has the highest percentage of plain platforms, but this might be a characteristic of this rock type which is very difficult to shape. These observations are confirmed in Fig 19 which show dorsal scar pattern and platform type for hornfels and dolerite. As can be seen, flakes with faceted platforms in hornfels have predominantly unidirectional or bidirectional scar patterns, which is consistent with blade/bladelet knapping methods. On the other hand, dolerite flakes with dihedral platforms show predominantly unidirectional convergent and centripetal/subcentripetal scar patterns, which are consistent with *Levallois*/discoidal reduction.

Again the Chi^2 analysis of the platform types in the three main rock types implies that the different categories appear in groups that are remarkably different, which might point to different strategies for each one of these three rock types [Dolerite: Deg. freedom:5, Chi^2: 1409.9, p(same): 9.7987E-303. Hornfels: Deg. freedom:5, Chi^2:251.67, p(same): 2.4077E-52].

**Fig 20:**
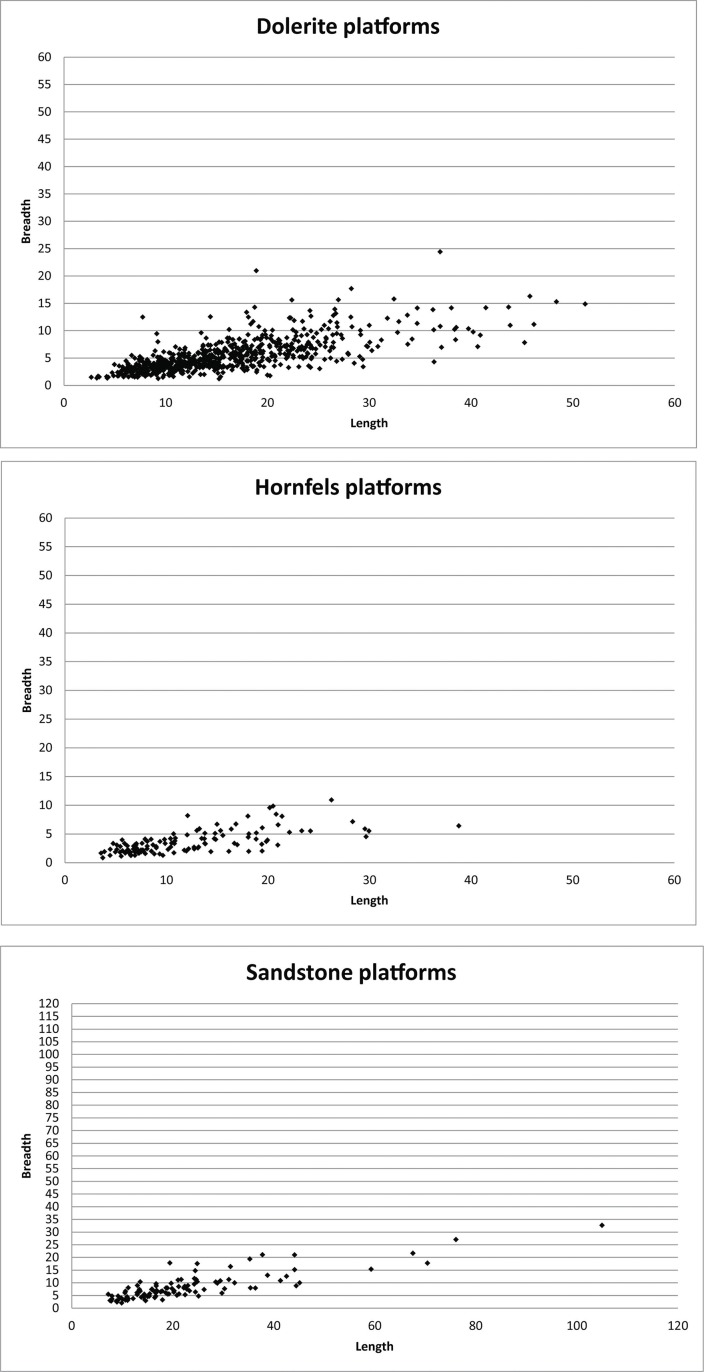
Typometrical distribution of platforms flakes by rock type in Sibudu, layer GR.

The knapping techniques applied to the blade production are difficult to determine. These are technological traits that have been mentioned in previous HP technological studies [6, 25, 33, 35]. It must be taken into account that hornfels and dolerite have very particular mechanical properties [56]; and that the macroscopic characteristics required to distinguish knapping techniques usually come from flint experimental programmes and, therefore, they should not be uncritically applied to hornfels and dolerite. Indeed, recently, even the qualitative approach to recognizing knapping techniques for flint has been questioned from a statistical analysis point of view [88]. I decided not to draw further conclusions about dolerite and hornfels knapping techniques until knapping experiments are done. The difficulties associated with determining knapping techniques of these two rock types are also evident when we look at the typometrical distribution of the platform flakes by rock type. As can be seen (Fig 20), dolerite has systematically bigger and broader platforms, which is probably due to its mechanical properties.

**Fig 21:**
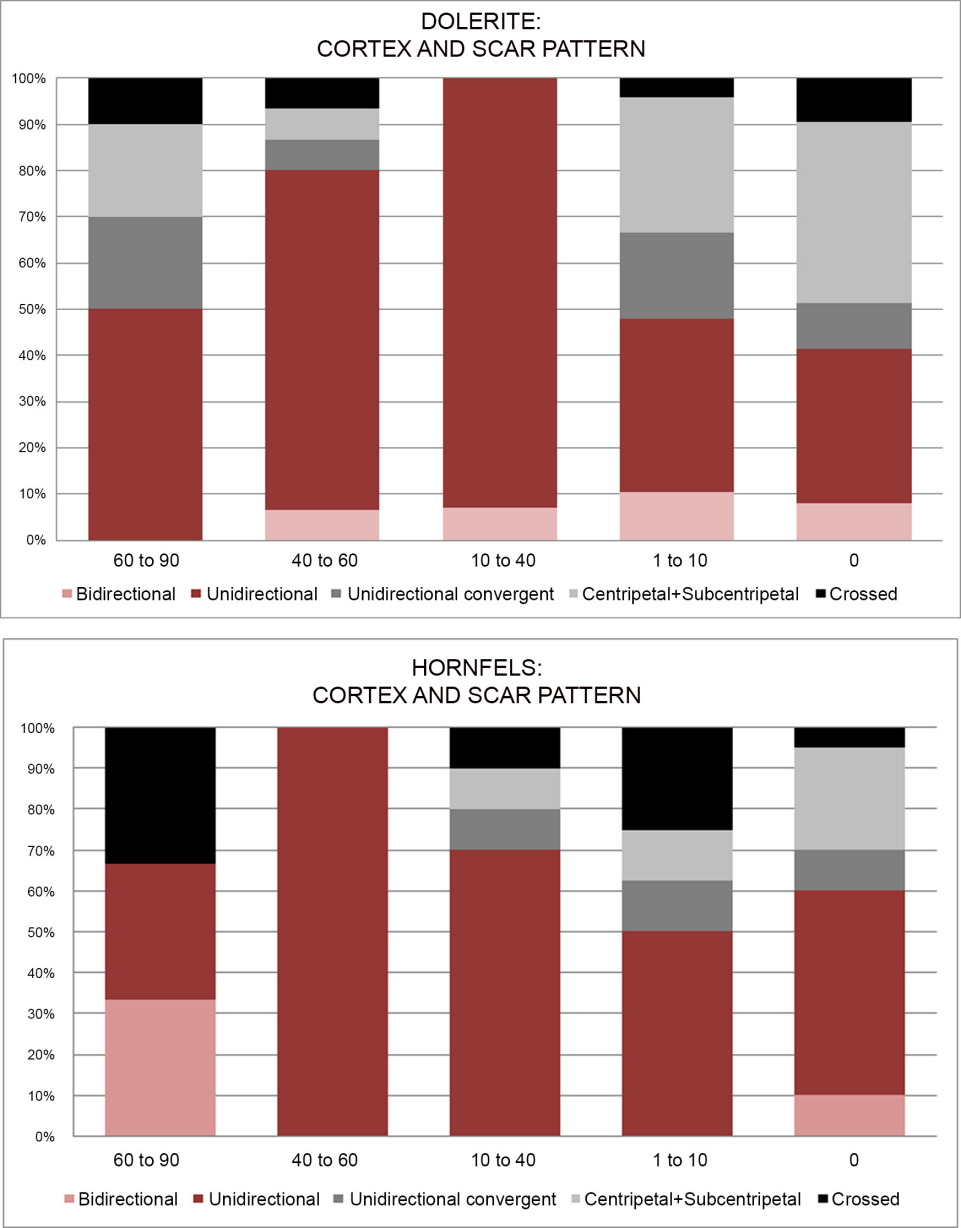
Cortex percentage and scar pattern for hornfels and dolerite in Sibudu, layer GR.

It is also useful to examine the relationship between cortex percentage and scar pattern for hornfels and dolerite. If we focus on the last stage of reduction, when the flakes have 1 to 10% or even no cortex, a different pattern for hornfels and dolerite can be observed (Fig 21). Dolerite has a more abundant percentage of centripetal and subcentripetal scars (>50%), whereas hornfels unidirectional scars are greater than 50%. In other words, it seems that for hornfels, even if there was flake production, the pursuit of blade (or elongated flakes) was valued more. Meanwhile for dolerite the opposite trend was observed (flakes were more important than blade production).

During the analysis of complete blanks I made the distinction between flakes and blades using technological criteria (*vid*. *supra*). As can be seen in Table 9 the percentage of blades in dolerite is greater than that of hornfels and sandstone, which goes against my previous statements about the frequencies of scar pattern, shape of blanks, platform types and cortex percentages (with dolerite having a greater tendency towards flake production than blade production, and the opposite trend for hornfels). However, the higher proportion of dolerite (technological) blades might be related to the fact that dolerite tends to fracture accidentally much less than hornfels [89] and sandstone; and that in this regard the frequencies of scar pattern and platform types are reliable.

**Table 9:** Sibudu, layer GR. Technological recognition of flakes and blades/bladelets for hornfels, dolerite and sandstone.

Technological recognition of flakes and blades/bladelets	Dolerite		Hornfels		Sandstone	
	N	%	N	%	N	%
Blade	265	24.58	234	22.63	213	21.7
Flake	813	75.42	800	77.37	768	78.3
**TOTAL**	**1078**	**100**	**1034**	**100**	**981**	**100**

#### What is the typometry of the complete blanks showing us?

**Fig 22:**
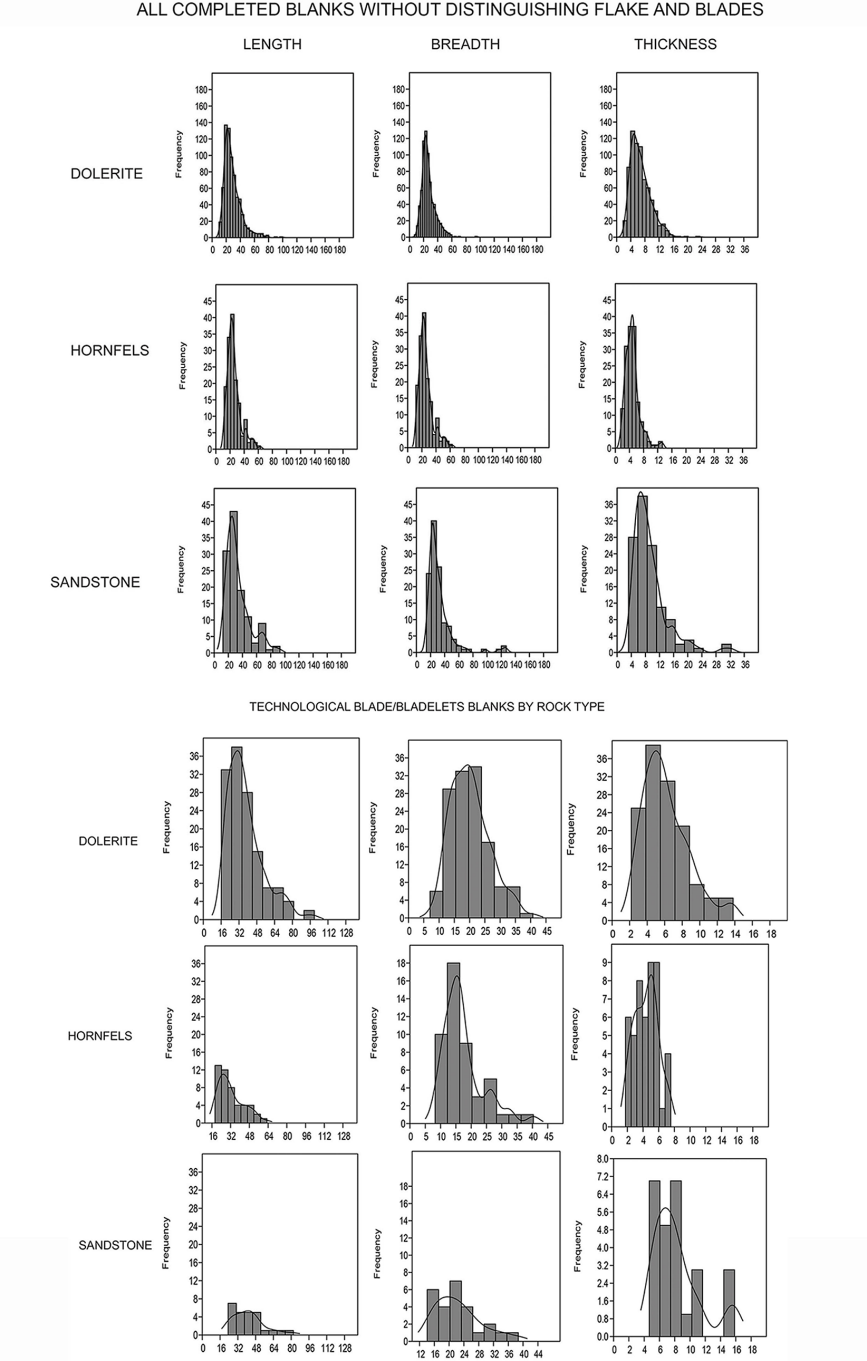
Sibudu, layer GR. Above: Histograms of all completed blanks without distinguishing between flakes and blades. Below: Histogram of only the pieces recognized as blade/bladelets technologically.

In regard to the typometry of all the blanks (without distinguishing by technological category, Table 10) (Fig 22) the Shapiro Wilk normality test shows how the three main measurements (length, breadth and thickness) for flakes do not show a normal distribution (Table 11). This is not surprising because, as I have explained in the methodology section, in a first analysis of the blanks without retouch I did not make the distinction between flakes and blades from a technological point of view (even if the technological distinction was recorded in my database) and I considered everything as flakes. As was highlighted in the previous sections, it is clear, from the qualitative characteristics of the cores, the core related by-products and the blanks without retouch that different knapping methods were involved inside each one of the management strategies of the different rock types. Therefore these overarching sets of data (Table 10) result from different knapping methods within the same rock type. For example, for dolerite we have seen so far that there probably was big blade production from prismatic cores, blade/bladelet production from HP cores, bladelet production from cores on flakes and flake production. Consequently, it is not surprising that the length or the breadth of the dolerite flakes does not have a normal distribution, because different populations of blanks come from different types of knapping methods.

**Table 10:** Sibudu, layer GR. Univariate statistics of length, breadth and thickness of all complete blanks in the three main rock types.

	Length Dolerite	Length Hornfels	Length Sandstone	Breadth Dolerite	Breadth Hornfels	Breadth Sandstone	Thickness Dolerite	Thickness Hornfels	Thickness Sandstone
**N**	710.00	150.00	119.00	710.00	150.00	119.00	710.00	150.00	119.00
**Min**	9.80	11.63	12.43	7.03	8.23	14.09	1.84	1.54	3.24
**Max**	99.69	63.01	93.03	95.71	48.87	127.23	23.49	13.30	32.28
**Mean**	28.75	25.55	33.54	26.85	21.54	32.58	6.79	4.85	9.29
**Std. error**	0.46	0.80	1.55	0.36	0.59	1.77	0.11	0.17	0.45
**Variance**	148.06	96.08	285.09	94.21	51.64	374.19	8.17	4.26	24.03
**Stand. dev**	12.17	9.80	16.88	9.71	7.19	19.34	2.86	2.06	4.90
**Median**	25.73	23.68	27.48	24.71	21.55	26.78	6.27	4.67	7.97
**Skewness**	1.71	1.35	1.41	1.50	0.76	3.06	1.14	1.54	2.12
**Kurtosis**	4.30	1.97	1.67	4.55	1.28	11.22	2.12	3.71	5.99
**Geom. mean**	26.68	23.96	30.16	25.34	20.37	29.25	6.25	4.48	8.36
**Coeff. var**	42.32	38.37	50.34	36.15	33.37	59.38	42.10	42.54	52.79

**Table 11:** Sibudu, layer GR. Shapiro-Wilk normality tests of length, breadth and thickness by rock type of all the blanks (without distinguishing by technological categories).

	Length Dolerite	Length Hornfels	Length Sandstone	Breadth Dolerite	Breadth Hornfels	Breadth Sandstone	Thickness Dolerite	Thickness Hornfels	Thickness Sandstone
**W**	0.8703	0.8927	0.8604	0.9057	0.9615	0.6698	0.9308	0.8904	0.8077
**p(normal)**	8.25E-24	5.19E-09	3.27E-09	1.57E-20	0.0003396	5.40E-15	1.38E-17	3.94E-09	3.53E-11

It is interesting to observe that hornfels and dolerite blanks have lengths and breadths with a very similar distribution (Fig 22, upper part). Dolerite is, however, always thicker in comparison to hornfels and sandstone; but this can be an intrinsic characteristic of the dolerite. As can be seen in (Fig 22-upper part), dolerite displays a clearly unimodal distribution while hornfels and sandstone produce a bimodal curve (which is clearer for sandstone).

It is also noteworthy that if we only take into account the blade/bladelet blanks in dolerite, hornfels and sandstone (using a technological distinction), the Shapiro Wilk test for length, breadth and thickness do not give a normal distribution (except for the thickness of hornfels blade/bladelets) (Fig 22-lower part and Table 12). This is probably because there are different groups of blade/bladelets that come from different blade knapping methods (core on flakes, bladelet cores from small nodules, big prismatic cores and HP cores). Indeed, the histogram of the blade/bladelet by-products is not giving unimodal distributions for either of the rock types (see for example length and thickness for dolerite or breadth for hornfels). A mixture of analyses was applied for the length of dolerite, hornfels and sandstone blade- blanks in order to explore the possibility that different sets of normal distributions were mixed within each one of these groups. As can be seen in Table 13 for the three rock types three groups of normal distribution were more coherent than one group, which supports the qualitative idea that more than one method was used for the production of blade/bladelets in each of the rock types.

**Table 12:** Shapiro-Wilk normality tests of length, breadth and thickness by rock type of all the blade and bladelets blanks (distinguished by technological criteria).

	Length Dolerite	Length Hornfels	Length Sandstone	Breadth Dolerite	Breadth Hornfels	Breadth Sandstone	Thickness Dolerite	Thickness Hornfels	Thickness Sandstone
**W**	0.8944	0.9049	0.9177	0.9697	0.8726	0.9218	0.9311	0.9727	0.8523
**p(normal)**	2.712E-08	0.0009057	0.03964	0.004342	9.163E-05	0.04953	3.813E-06	0.3208*	0.001577

The cases which show normality are highlighted with an asterisk.

**Table 13:** Mixture analysis for the length of dolerite, hornfels and sandstone blade/bladelets blanks.

**Dolerite: three groups. Log: -412.4. Akaike:837.5**		
Prob	Mean	Stdev
0.60111	32.658	7.9396
0.27392	56.742	17.059
0.12496	21.708	1.2309
**Hornfels: three groups. Log:-127. Akaike IC:268**		
Prob	Mean	Stdev
0.318	45.786	7.8336
0.27545	21.847	1.8279
0.40655	29.014	3.531
**Sandstone: three groups. Log:-74.9. Akaike IC: 166.2**		
Prob	Mean	Stdev
0.16138	66.645	10.514
0.57815	41.666	5.5817
0.26046	25.967	1.7993

### The Retouched Blanks

#### What is the general representation of retouch blanks by rock type?

When the retouched pieces are examined by rock type, the percentages suggest a different distribution from what we have seen so far in other technological groups (cores, core related by-products, etc.) In this case dolerite and quartz are the most retouched rock types, followed by hornfels. In addition, there are some retouched pieces in quartzite and CCS (Table 14).

**Table 14:** Formal tools and retouched pieces in layer GR according to rock type.

	Dolerite		Hornfels		Quartz		Quartzite	CCS
	N	%	N	%	N	%	N	N
Denticulate	1	1.20						
End-Scraper	2	2.41						
Borer			1	1.45				
Notch			2	2.90				
Micronotch					8	9.09		
Burin			2	2.90				
Strangulated piece	1	1.20	2	2.90				
Simple retouch flake	3	3.61	2	2.90	1	1.14		
Simple retouch blade	6	7.23	5	7.25				1
Marginal retouch flake	1	1.20	3	4.35				
Ind. Retouch (simple retouch)	5	6.02	5	7.25	2	2.27		
Ind. Segment	3	3.61	10	14.49	3	3.41	1	
S1	13	15.66	8	11.59	2	2.27		
S2	8	9.64	2	2.90	1	1.14	1	
ST2	1	1.20						
ST3	1	1.20						
ST4			1	1.45				
Tr1	1	1.20	1	1.45				
Tr2	1	1.20	1	1.45				
DT1	1	1.20	1	1.45				
T2			1	1.45				
Indeterminate Truncation	13	15.66	1	1.45				
Rectilinear truncation	1	1.20						
Backed bladelet <12mm	1	1.20	18	26.09				
Ind. Backed	17	20.48			6	6.82	1	
Bifacial fragment or complete pieces	3	3.61	3	4.35	65	73.86	1	
TOTAL	**83**	**100.00**	**69**	**100.00**	**88**	**100.00**	4	1
% by rock type	**33.88**	** **	**28.16**	** **	**35.92**	** **	**1.63**	**0.41**

**Fig 23:**
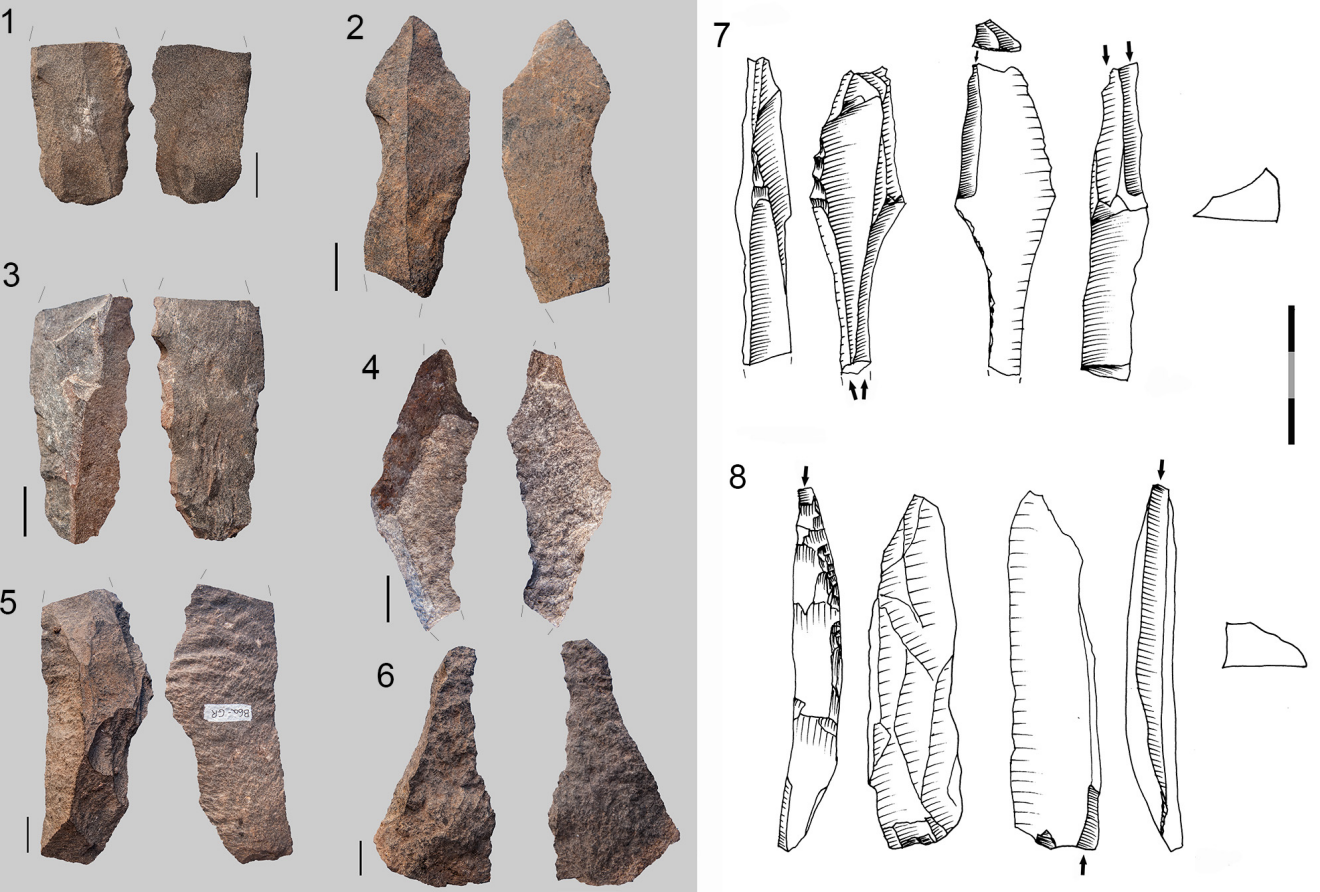
Different morphotypes from layer GR, generally referred to as ‘domestic tools’ in this paper. 1, 3 and 6 Retouched blade on hornfels and dolerite. 2 and 4 Strangulated blade on hornfels and dolerite. 5. Pieces with macrotraces on dolerite. 7 and 8 Burins on hornfels. Piece number 8 is a burin made on false semicrest from a big prismatic blade dolerite core.

**Fig 24:**
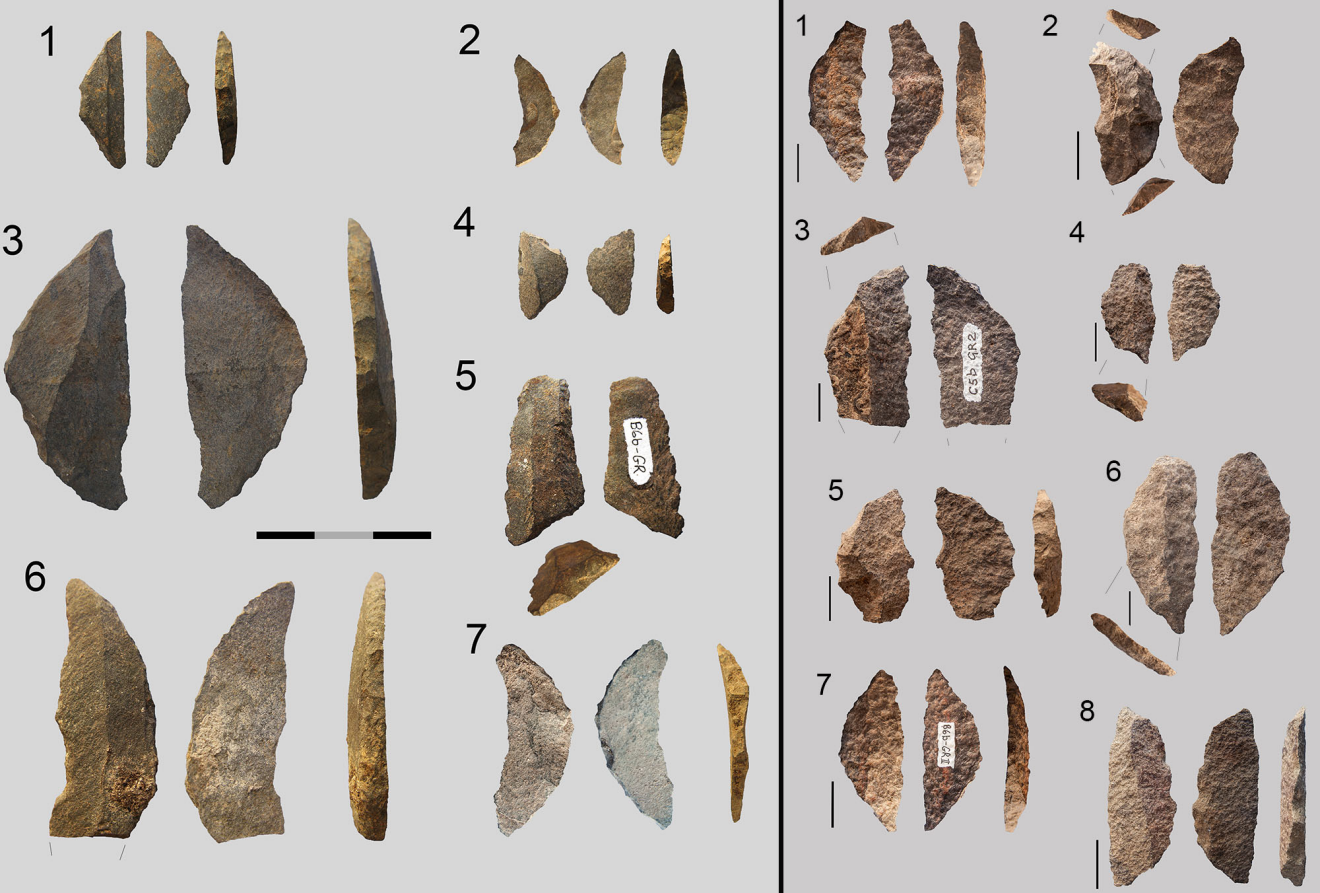
Backed tools in hornfels (left) and dolerite (right). For hornels on the left: 1. Trapeze. 2,4,3,6 and 7. Segments. 5. Single truncation. For dolerite (on the right): 1,5,7 and 8.Segments. 2, 3, 4 and 6. Truncations.

**Fig 25:**
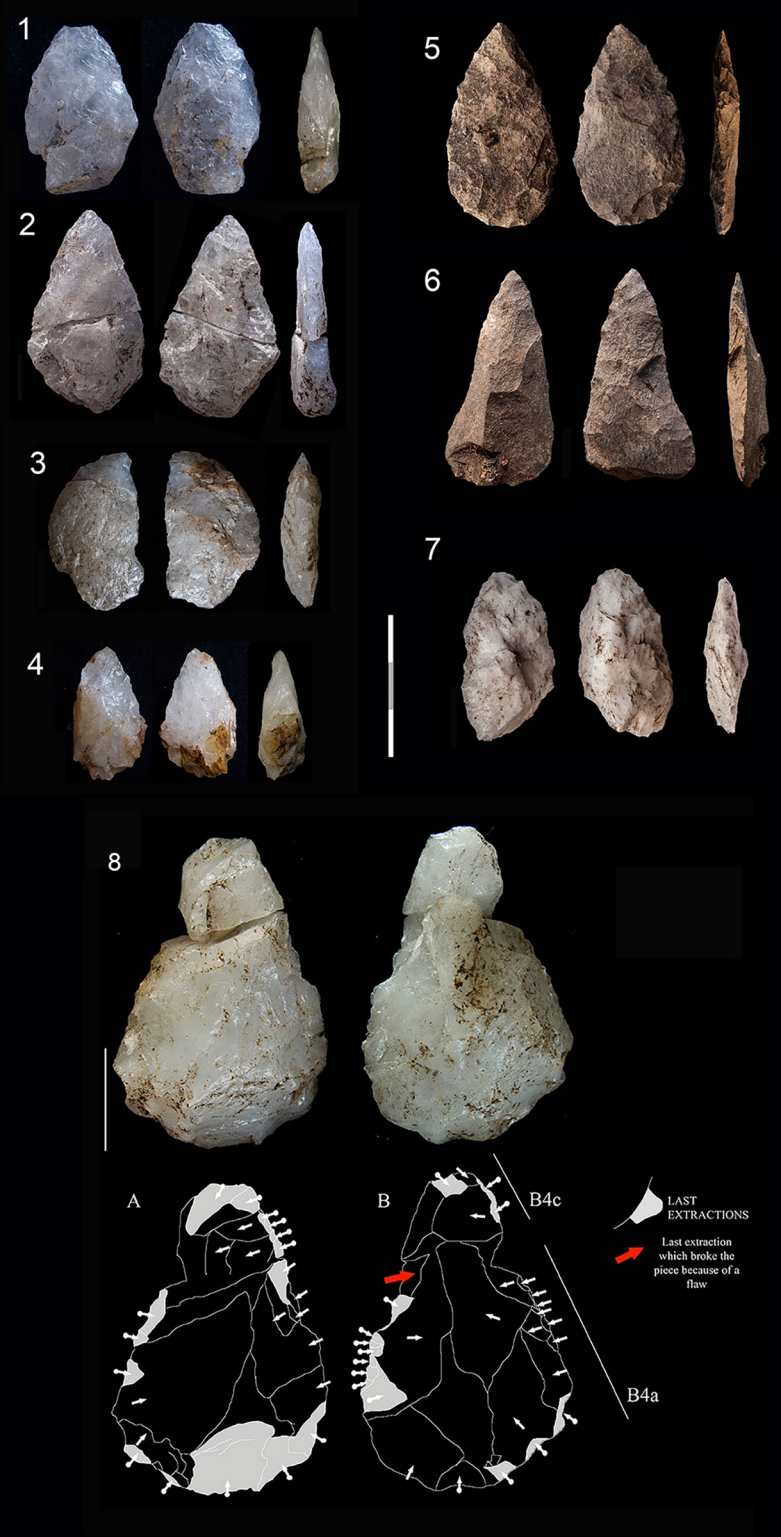
Bifacial pieces in GR layer. 1,2,3,4 and 7 on quartz. Pieces # 2 and 8 are refittings. 5 and 6 on hornfels.

**Fig 26:**
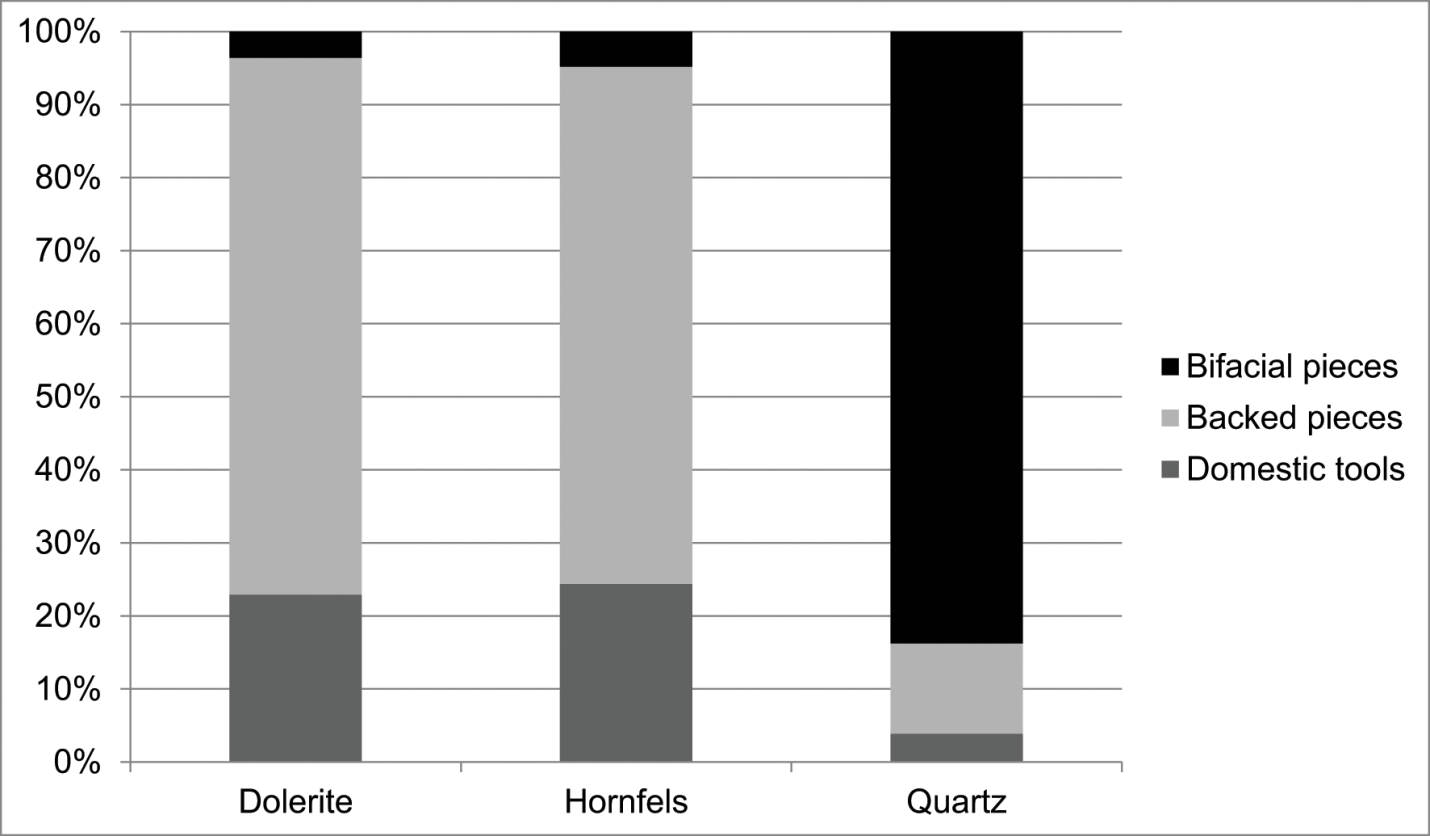
Representation of domestic tools, backed pieces and bifacial pieces for the three main retouched rock types in GR layer.

It is clear that sandstone (the third most common rock type represented in this layer Fig 3) is not retouched at all. This fact probably means that there was a huge amount of rock knapped (and potentially used) but very seldom retouched. In order to give an idea of the general trends for the management of retouched pieces in the three main rock types I have grouped the morphotypes into three big categories: domestic tools (with this category I refer to tools shaped by simple retouch such as end-scrapers, borer, strangulated piece, notch and burin) (Fig 23), backed tools (Fig 24), and bifacial pieces (Fig 25). I acknowledge that these categories are completely artificial groups. Nonetheless, this division of morphotypes (Fig 26) simplifies Table 14 considerably and, thus, shows eloquently that in fact hornfels and dolerite have a very similar pattern of morphotype percentages, with backed pieces around 60–70%, followed by domestic tools (3–5%) and a very minor percentage of bifacial pieces. In contrast, quartz is mainly directed toward the production of bifacial pieces, as stated in a previous specialized publication around this HP morphotype [19].

#### What types of blanks were preferably retouched?

It is worth noting that the preferred blanks for conversion to retouched pieces are always blades for backed pieces and domestic tools, and flakes for bifacial pieces. In all cases where I could identify (technologically) the type of blank, it was always a blade for backed pieces. Amongst the domestic tools there are some flakes used as blanks, but remarkably few (n = 8, 6 in dolerite and 2 in hornfels). However, for the bifacial pieces on quartz most of the blanks, when they can be recognized are, on the contrary, on flakes [37]. Therefore, this reinforces the idea that most of the flake production for hornfels, dolerite and sandstone was performed without the necessity for shaping or resharpening (retouch).

#### What type of morphotype representation do we have within the category backed pieces?

For the three main rock types it is clear that segments are the most frequent backed pieces (Table 14, Fig 24), followed by truncations (see Fig 5 to understand the subtypes distinguished in this study). It would be interesting in the future to investigate whether there is a functional distinction between these two subtypes of morphotypes.

#### What does the typometry tell us about the backed pieces?

It is noteworthy that the length and breadth of backed pieces show normal distribution (Table 15). This is remarkable because previously it has been shown that the blade/bladelet (without retouch) blanks do not show a normal distribution and probably there were at least three groups of blade production (after a mixture of analyses, *vid*. *supra*). Consequently, there was a selection of blanks for backing. Indeed, one of the means proposed by the mixture analysis of dolerite length (mean 32.66) matches quite well with the mean of the length of dolerite backed pieces (mean: 34.68) (cf. Tables 13 and 15).

**Table 15:** Shapiro-Wilk normality tests of length, breadth and thickness for dolerite and hornfels backed pieces in GR. Quartz was not included as it has a really small number of cases.

	Length Dolerite	Length Hornfels	Breadth Dolerite	Breadth Hornfels	Thickness Dolerite	Thickness Hornfels
**W**	0.9458	0.8584	0.9239	0.9412	0.7371	0.816
**p(normal)**	0.5762*	0.09216*	0.005128*	0.1076*	2.635E-08	3.439E-05

Cases which show normality have been highlighted with an asterisk.

Furthermore, the standard deviation is less than half the value of the mean for length and breadth in dolerite and hornfels (Table 16), which means that the variability for these two parameters is low. For thickness this rule also applies, but it is less pronounced.

**Table 16:** Univariate data for dolerite and hornfels backed pieces in GR.

	Length dolerite	Length hornfels	Breadth dolerite	Breadth hornfels	Thickness dolerite	Thickness hornfels
**N**	12.00	9.00	46.00	29.00	52.00	36.00
**Min**	24.53	15.77	8.90	8.20	2.50	2.05
**Max**	44.48	53.36	30.66	22.97	16.50	11.68
**Mean**	34.68	30.11	16.60	13.85	5.47	4.41
**Std. error**	1.96	3.39	0.57	0.68	0.31	0.33
**Variance**	46.28	103.62	14.95	13.43	5.08	3.81
**Stand. dev**	6.80	10.18	3.87	3.67	2.25	1.95
**Median**	34.33	28.67	15.91	13.76	5.13	3.89
**Skewness**	0.09	1.42	1.24	0.67	2.88	2.00
**Kurtosis**	-1.31	3.84	3.21	0.66	11.49	5.03
**Geom. mean**	34.06	28.74	16.20	13.39	5.15	4.10
**Coeff. var**	19.62	33.80	23.29	26.47	41.21	44.22

**Fig 27:**
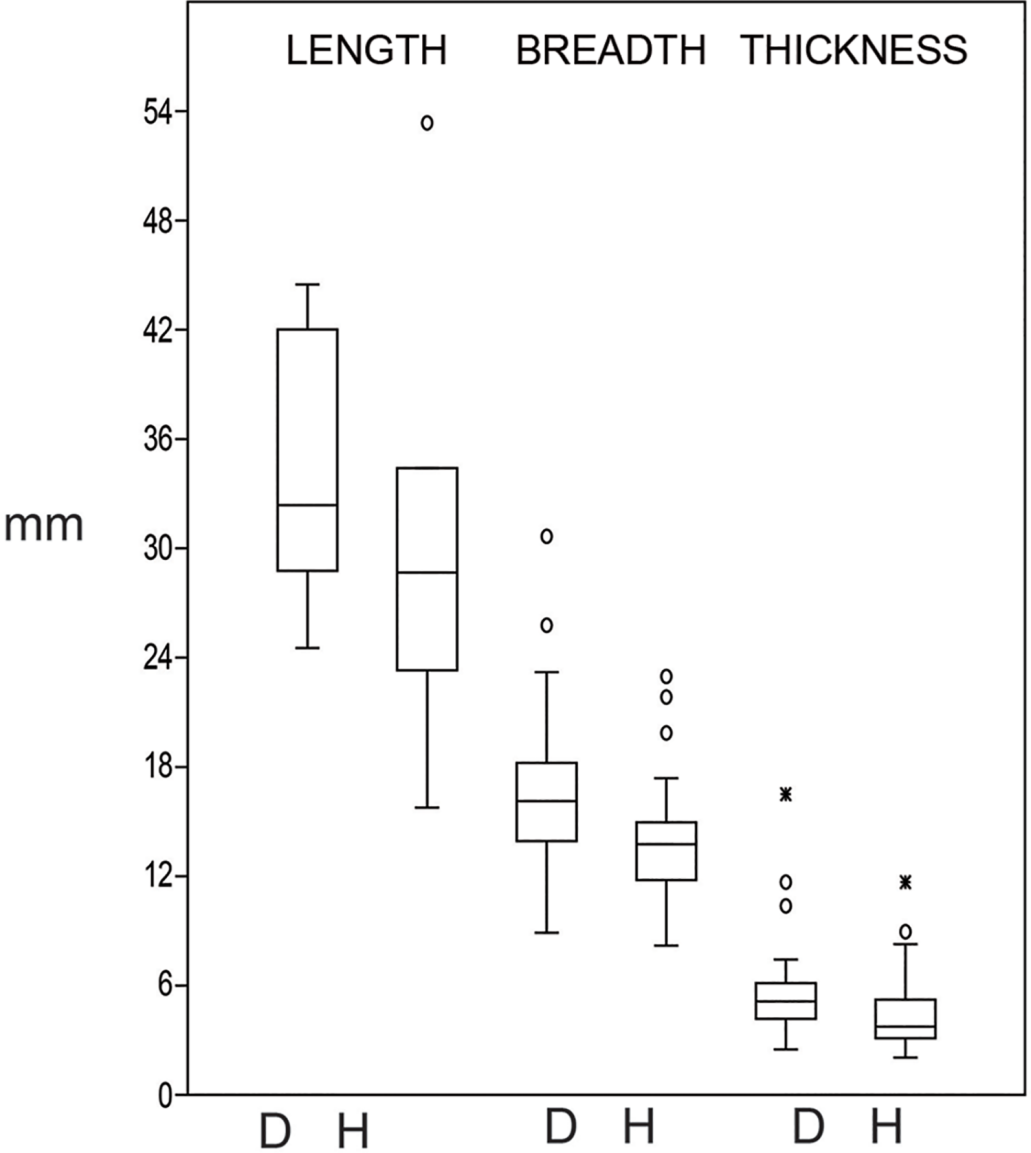
Box-plot of length, breadth and thickness of dolerite and hornfels backed tools.D = Dolerite, H = Hornfels.

In addition, a F test performed for breadth and thickness showed that there are not significant differences in breadth parameter for dolerite and hornfels (see Table 17 and Fig 27). I used the breadth to compare them as most of the pieces can give maximal breadth but, on the contrary, many of these pieces are broken in their distal or proximal part and, therefore, length it is not so suitable to compare them.

**Table 17:** F test and T test for breadth of backed pieces in hornfels and dolerite from layer GR.

TESTS				
**F:**	1.1132		p(same):	0.77579
**t:**	3.0628		p(same):	0.00307
**Uneq. var t**	3.1007		p(same):	0.002902
**Permutation t test (N = 9999):**	p(same):	0.0027		

I have not compared hornfels and dolerite backed pieces to quartz ones because in GR there are very few backed pieces in this rock type and none of them is completed. However, the backed pieces on quartz are notably smaller, as was already noted in GS layer (where there is a bigger sample, see [37] and Fig 15 within it) and also in PGS [42].

#### How is the bifacial production carried out?

The GR quartz bifacial points are made on flakes and are generally unstandardized in shape and size [19]. Detailed technological analysis of GR points revealed a short reduction sequence without successive thinning stages (this is mainly due to the size of the quartz pebbles, ~5-10cm). The brevity and simplicity of the point reduction sequence may partly reflect the high risk of knapping accidents with quartz. In a previous work four phases for the manufacturing of these points were hypothesized:

1. Selection of optimally-sized flake blanks made from discoidal or unprepared cores; 2. Minimal thinning whereby unfinished blanks display attributes of either unifacial reduction (knapping one flake face initially, then the other) or a bifacial strategy with alternating blows to the two faces, a step that conflates Callahan’s [90] thinning stages 3 and 4; 3. Shaping of the blank to a refined preform, probably made with a combination of hard mineral hammer and soft organic hammer, resulting in regular scars; 4. Final shaping of the preform and finished product, possibly with a soft organic hammer and pressure flaking.

In this new detailed study of the technology of GR I found seven more bifacial pieces that must be added to the inventory published in de la Peña et al. [41]. Five of these pieces are quartz and two are hornfels. Furthermore, one of the new pieces (a quartz base) refits with a tip from one of the points published in [19] (Fig 25 #2).

#### The quartz retouch production for the non bifacial pieces

Quartz bladelet technology is present in GR first from freehand prismatic bladelet cores and then from bipolar cores. The production was focused on obtaining tiny bladelets to be used without retouch, or to be converted into small backed pieces or other morphotypes (such as small single or double notches).

## Discussion

The GR technological study has shown a great variety of knapping methods. The hallmarks of the GR lithic technology are:

▪ Different varieties of blade knapping methods (from HP cores, big blade prismatic cores, core on flakes).
▪ A strong component of flake production that for hornfels, dolerite and sandstone was not retouched.
▪ The production of backed tools of different size ranges and shapes for hornfels, dolerite and quartz. Segments are the most frequent morphotype.
▪ Quartz nodules are maximized through reduction of freehand bladelet prismatic cores, then bipolar cores.
▪ The production of bifacial pieces on quartz.
▪ The use of coarse grained rock types such as sandstone or dolerite.

After the in depth study of the management of the different rock types it is clear that different objectives and strategies were developed depending on the rock type at hand, as postulated in the methodological analysis (null hypothesis, see Methodology section). Moreover, it seems that the low numbers of domestic tools is probably owed to the fact that a lot of activities were performed with non-retouched blanks, whereas shaped blanks (formal tools) such as backed implements or bifacial pieces were probably devoted to hunting strategies, and this suggestion is supported by previous research on Sibudu’s HP backed tools [41–44].

This technological analysis complements the recent study of Soriano et al. [35] because different blade knapping methods have been highlighted. Moreover, in the present analysis, flake production has been demonstrated as highly significant. Finally, the representation of quartz, even if it is not the most frequently used rock type, has been demonstrated as substantial because of the high representation of retouched pieces in this mineral (bifacial pieces and backed pieces) and because of the strategy of maximizing the quartz pieces through reduction by bipolar knapping.

Once the description of the main technological trends of this so-called HP assemblage has been performed, the questions lacking an answer are: how does this technological description relate to other assemblages also defined as HP? More importantly, what is the *technological* idiosyncrasy of the HP?

In order to discuss the variability within HP assemblages it is appropriate to point out that there are differences and similarities between and within HP assemblages already published from other sites.

Five of the main technological knapping methods highlighted for GR have been also detected in other HP sequences; let us first review the evidence:

The so called HP cores described in Wurz [25] and in Villa et al. [34] are present in hornfels and dolerite in GR, but they are not the only blade knapping methods for blade blanks in HP assemblages. As pointed out previously prismatic blade production and cores on flakes play an important role in the blade production in the GS [36] and GR layers of Sibudu.

The flake production detected in this study coincides with the discoidal and *Levallois* flaking methods described for the most recent layers of other HP contexts such as Klasies River [25], Rose Cottage (layers ETH and SUZ) [33], Diepkloof (layers ascribed to the ‘Late HP’)[6] and Klipdrift (layers PBC, PBA/PBB, PAZ, and PAY, even though these discoidal cores occur throughout the HP sequence)[39]. The increase of flake production could be a temporal marker towards the end of the HP technocomplex.

The bipolar knapping production for quartz in GR seems to be a recurrent strategy for the maximum reduction of this rock and it also supposes continuity with GS and PGS within the HP Sibudu sequence [37, 84]. The use of bipolar knapping has been interpreted here as the most efficient method of reducing quartz small cores and/or nodules. Quartz was probably highly valued as a rock because of its mechanical properties and its relative scarcity in the Verulam area. Some reasons for its choice are difficult to elucidate. Although abundant in this area, the physical occurrence of quartz is only in small pebbles (5-10cm). Thus, this knapping strategy was a solution to maximize the production of bladelets or small flakes, as has been emphasized in other geographic areas and periods [91, 92]. Bipolar knapping has been also observed in other HP contexts of southern Africa. Wurz [25] mentioned *outils écaillés* in the HP sequence of Klasies River from the Singer and Wymer 1967/8 excavations. She interpreted them as products of extended core reduction and not as tools. Mackay [32] pointed out that in Diepkloof bipolar cores are common in the >74, 70–65, 65–62 and 62–60 ka layers. Meanwhile at Klein Kliphuis bipolar cores are common between 62 and 60 ka.

As stated in the introduction backed pieces are the principal typological marker in the HP [26]. In this study I have given a specific classification for these types of pieces (Fig 5). They may point to different functional solutions [16] and their description and refined definition can help us in the future to understand better functional variation in the HP [42]. Moreover, they might be pointing out the imposition of attributes of style [26] and maybe regional variations within HP. For example, the obliquely backed points constitute the main backed morphotype in the HP layers of Rose Cottage [17, 38], whereas in Diepkloof different backed morphotypes are predominant, such as the geometric backed tools in the early HP or the large backed pieces in the MSA type ‘Jack’ [6]. In Pinnacle Point the HP is identified in layer ‘Dark Brown Compact Sand’ (DBCS) (with three OSL dates ranging 58±4 to 65±4 ka) by backed tools and notched tool forms. However, this site has an older layer, ‘Shelly Ashy Dark Brown Sand’ (SABDS) with a weighted mean OSL date of 71.1±2.3ka, also with backed pieces; which is presented as an ‘unrecognized advanced stone tool technology’ (*sic*) within the MSA [93]. The main difference stated between these two Pinnacle Point layers (DBCS and SABDS) is, first, the typometry/length-width ratio of the backed pieces and, secondly, SABDS backed pieces are statistically different from the Klasies River HP backed pieces. However, it must be stressed that there is a great deal of morphological and typometrical variation in HP backed pieces [42]. This was highlighted in Sibudu’s GS layer where quartz and dolerite and hornfels backed pieces demonstrate morphological and typometrical variation, see [37] and Table 12 and Fig 15 within it; and this variation also occurs in GR (although is not provided because most of the quartz backed pieces are incomplete and have multiple fractures). In Sibudu’s GR layer we see a predominance of segments, but other morphotypes are also represented (see Fig 5 and Table 14).

Bifacial pieces are also a common characteristic in both the Sibudu and Diepkloof HP sequences [6]. However, the GR and GS bifacial pieces are mainly made on quartz [41] whereas the pieces from Diepkloof are made on silcrete. It is difficult to compare these two assemblages of bifacial points as most of the pieces from Sibudu seem to be failures or fragments produced during the knapping reduction sequence and there are very few examples which appear finished [41]. In other words, it is difficult to visualize a standardized pattern from their shape.

Apart of these common and important features it must be highlighted that none of the techno-typological models mentioned in the introduction entirely works for Sibudu, even if there are remarkable technological trends in common. One of the most evident reasons is simply the representation of formal tools. For example, the bifacial production at Sibudu appears mainly in GR (the most recent HP layer in this sequence) and in GS [36]. If the HP from Sibudu corresponds to the so-called ‘classic HP’ *sensu* Porraz et al. [6], it is quite contradictory that the bifacial production appears in its most recent layers, whereas at Diepkloof this is presented as one of the hallmarks of the early phase of HP (*vid*. *supra* to compare the chronology for these layers), together with *pièces esquillées* (for this site interpreted as tools, see [46] but see [32]) and the truncated pieces. It could be argued that the bifacial pieces and different point production at Sibudu and Diepkloof constitute variations within the HP technocomplex which, indeed, seems more than likely.

Another reason for the discrepancy could be that the *pièces esquillées* in Sibudu have been interpreted as bipolar cores [36, 37, 72]. In the Sibudu sequence they are represented in similar percentages in all three layers [72], whereas in Diepkloof they seem especially abundant in the early HP phase. In Rose Cottage the bipolar knapping is prominent only in the post-HP layers [33].

**Fig 28:**
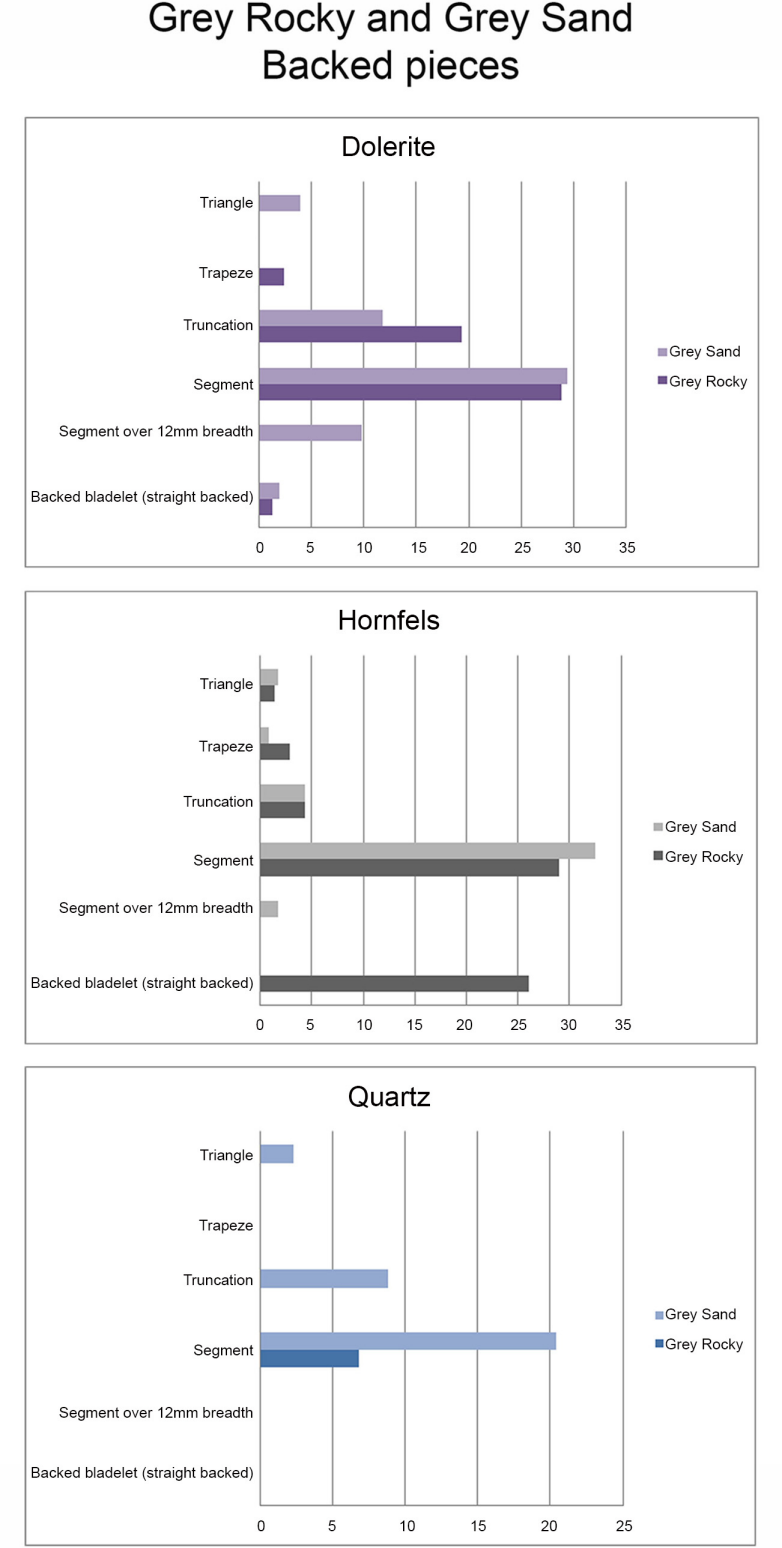
Comparison of percentage of GR and GS backed pieces.

**Fig 29:**
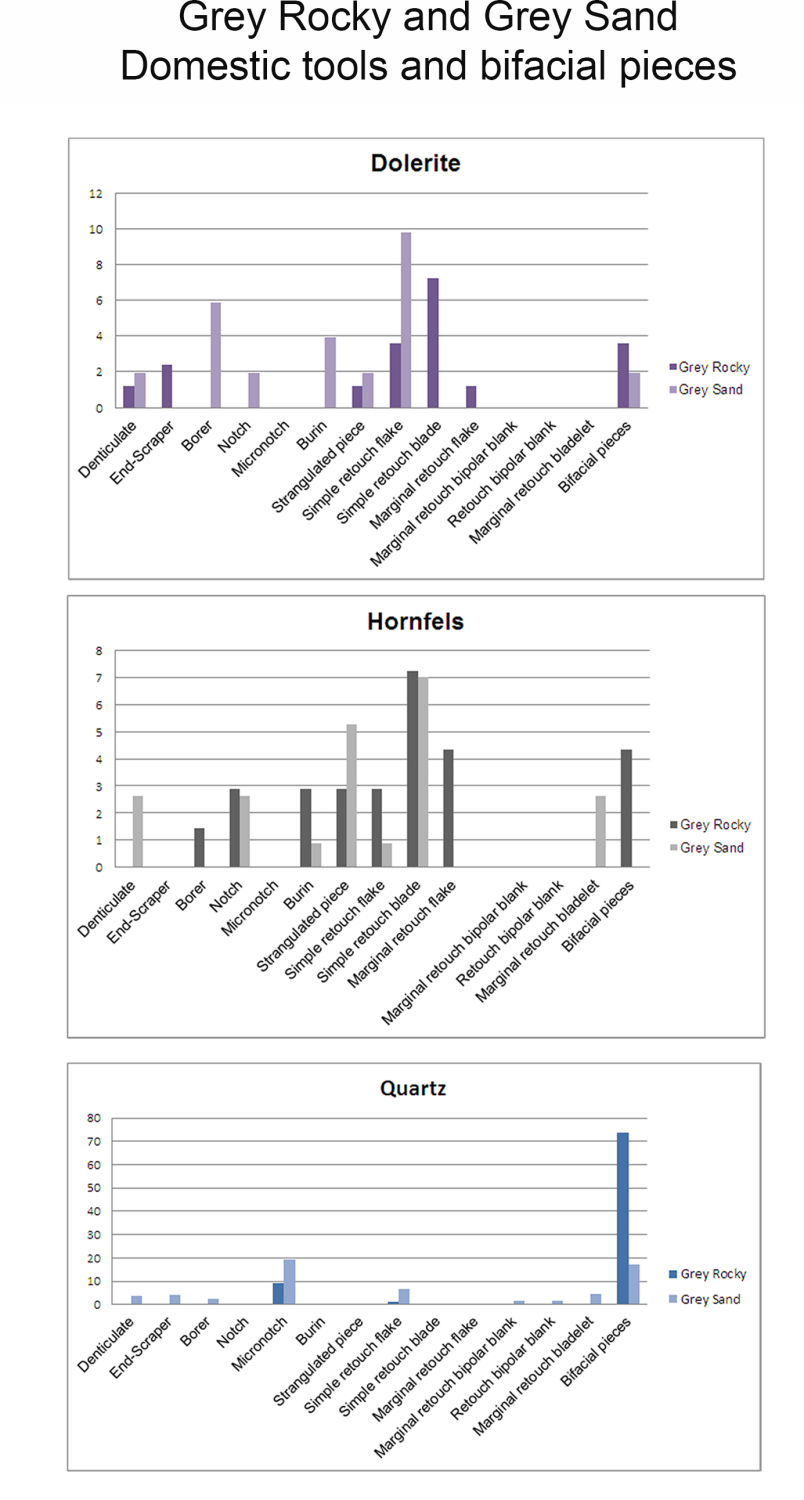
Comparison of percentage of GR and GS domestic tools.

A third technological characteristic which does not coincide with Diepkloof or with Rose Cottage is the deterioration of the blade production pointed out in these two sequences [6, 33]. In Sibudu I could not detect any such trait in GR, Indeed, in GR the blade knapping methods seem very similar to the ones documented in GS [36]. The variations between these two layers seem mainly functional, expressed in GR by the abundance of flake blanks or the percentage of formal tools (Figs 28 and 29). Moreover, to evaluate knapping performance in these terms seem to me highly subjective.

A fourth Sibudu characteristic which does not coincide with the other HP sequences is the main knapping method. For example, at Sibudu the blade production does not only come from HP cores (as in Klasies River [34]), even though this type is represented. Moreover, prismatic blade production seems more prominent. We have also seen, from the study of Rose Cottage, that this characteristic can be adapted to the raw material available locally [33].

As pointed out in the introduction these discrepancies between sites might come from different reasons: different diachronic variations, functional variations or maybe different regional variations (differences in style) within what has been recognized traditionally and typologically as HP [5, 94]. Furthermore, it should be remembered that some of the technological characteristics pointed out here coincide with other technological developments within the MSA [95]; for example the flake production (*Levallois* and discoidal), the bifacial pieces, the big prismatic blade production, the importance of local rock types and the bipolar knapping.

After all these comparisons the question is, once again, what defines HP? Moreover, what is HP? As highlighted in the introduction, HP has been defined as an industry, MSA entity, horizon marker, technocomplex or a techno-tradition. Moreover, even if an important effort to define the lithic technology associated with it has been made lately, the truth of the matter is that it is still mainly recognized by the typology. I do not recall any South African MSA sequence named HP only by its technology when there is a lack of backed pieces. However, with some of the technological trends highlighted here and in previous works (such as HP core reduction sequence, big prismatic blade production, core on flakes, flaking methods, bipolar knapping, the importance of backed morphotypes and the presence of a bifacial reduction sequence) maybe in the future we can go further in order to recognize this technocomplex uniquely by its technology. In other words, the technological trends highlighted at the beginning of this discussion, and this specific technological analysis, based on inter-site comparisons, supports the contemplation of HP as a technocomplex.

Recently Mackay et al. [96]:36 highlighted various broad inter-regional consistencies in HP assemblages that give them a unity: the basic forms of implements, the production of small blades using similar flaking systems, assemblage sizes and the on-site reduction of cores and production of implements.

The new technological studies are refining our understanding of this HP technocomplex (as this paper has tried to contribute). The fact that GR shares technological characteristics, but also shows some remarkable discrepancies, might be pointing out regional variation of this technocomplex. Moreover, when we want to compare different assemblages, the difficulty comes with the current chronological debate which makes this task highly problematic. As we are discussing variability between different sites it must be pointed out that it is extremely contentious to correlate HP assemblages chronologically because, as explained earlier, with the current chronological models [3] vs [47] there is a big difference between a technocomplex considered as a ‘short lived’ cultural phenomenon (5ka) (in ‘prehistoric’ terms) as opposed to a technocomplex lasting 45-50ka. Following the Diepkloof model [6, 47] GR lithic technology should be compared with the Intermediate or Late phase of HP. Moreover, with the current discussion around the chronology of HP and SB, it is difficult to know which climatic factors influenced (and if effectively they had an influence) the appearance, development and disappearance of the HP. This is quite a big issue because, as pointed out before, the majority of interpretations of the HP have been made within an ecological paradigm.

In any case, GR technology demonstrates a specific moment in time within the HP technocomplex and, as specified before, probably a regional expression. In this sense it is representative of this area at this specific period which, following Jacobs and Robert [49], is 61.7±2 ka, obtained from single grain optically stimulated luminescence on sediment from GR2. The Sibudu GR layer adds interesting input to the interpretation of the variability of HP in this eastern part of southern Africa. After the analysis of the GS layer [36, 37] and this new GR study, it is evident that most of the knapping methods represented in GS appeared also in this more recent layer GR. Thus, there is an evident continuity in this regard. Moreover, the same groups of formal tools are represented with slight variations. Therefore, there is an evident continuity in terms of technology and the variations of formal tool percentages could be interpreted in terms of functionality (Figs 28 and 29).

The detailed technological analysis of layer GR has shown the importance of blade production but also of flaking methods in coarse grained rock types. Moreover, new strategies of bifacial production and microlithism were really important (as the management of quartz has shown). Thus, GR lithic technology shows a really versatile example of reduction strategies that were highly influenced by the characteristics of the rock types. After this detailed analysis the challenge is to evaluate how the HP changes through time and why. This is also important for understanding the putative change from HP to the so-called post-HP [97, 98].

The GR lithic industry represents a diverse management of rock types; several strategies highlighted in this paper coincide with those in other sequences in southern Africa. In contrast, other characteristics, such as the management of quartz, seem site specific within the same technological tradition. However, the general characteristics pointed out by Mackay [96] for HP (the basic forms of implements, the production of small blades using similar flaking systems, assemblage sizes and the on-site reduction of cores and production of implements) can be found in all Sibudu HP layers. On the one hand, the technological studies from sites like Klasies River, Diepkloof, Klein Kliphuis, Rose Cottage and Sibudu, demonstrate that there are common technological trends during the late Pleistocene, but they still need to be properly circumscribed chronologically. On the other hand, HP characteristics such as the bifacial production in quartz are reminiscent of production in some SB or pre-SB industries (e.g. the so-called Pietersburg) and the flake production or the prismatic blade production described here could be a point in common with pre-SB and post-HP industries [99].

In this paper I have inquired into the technological characteristics of a so-called HP assemblage, and compared it in order to see whether the ‘technocomplex’ definition resists this comparison. I believe that a detailed technological and comparative study of HP and other MSA industries can attenuate the character of extreme originality presupposed for HP without removing its technical personality. Moreover, the fact that typologically this industry has attracted such attention might have created a tendency to highlight its unique status within the MSA [100]. In the same sense that studies from other archaeological branches, such as archaeobotanical remains, are pointing out advanced technologies within other phases of the MSA [101, 102], technological studies can demonstrate that it is the entire MSA, rather than simply the HP, that is exceptional.

## Supporting Information

S1 File The blanks without retouch database. Table A. (XLSX)
